# Physiological Changes and Transcriptomic Analysis throughout On-Tree Fruit Ripening Process in Persimmon (*Diospyros kaki* L.)

**DOI:** 10.3390/plants12162895

**Published:** 2023-08-08

**Authors:** Tania Dorta, Francisco Gil-Muñoz, Fany Carrasco, Elena Zuriaga, Gabino Ríos, Manuel Blasco

**Affiliations:** 1 Valencian Institute for Agricultural Research (IVIA), Road CV-315 Km 10.7, 46113 Valencia, Spainrios_gab@gva.es (G.R.); 2 CANSO, Avenue Cooperativa Agrícola Verge de Oreto, 1, 46250 L’Alcudia, Spain

**Keywords:** ethylene, transcriptional regulation, fruit ripening, softening

## Abstract

The involvement of effectors and transcriptional regulators in persimmon fruit maturation has been mostly approached by the literature under postharvest conditions. In order to elucidate the participation of these genes in the on-tree fruit maturation development, we have collected samples from seven persimmon germplasm accessions at different developmental stages until physiological maturation. This study has focused on the expression analysis of 13 genes involved in ethylene biosynthesis and response pathways, as well as the evolution of important agronomical traits such as skin colour, weight, and firmness. Results revealed different gene expression patterns, with genes up- and down-regulated during fruit development progression. A principal component analysis was performed to correlate gene expression with agronomical traits. The decreasing expression of the ethylene biosynthetic genes *DkACO1*, *DkACO2,* and *DkACS2*, in concordance with other sensing (*DkERS1*) and transduction genes (*DkERF18*), provides a molecular mechanism for the previously described high production of ethylene in immature detached fruits. On the other side, *DkERF8* and *DkERF16* are postulated to induce fruit softening and skin colour change during natural persimmon fruit ripening via *DkXTH9* and *DkPSY* activation, respectively. This study provides valuable information for a better understanding of the ethylene signalling pathway and its regulation during on-tree fruit ripening in persimmon.

## 1. Introduction

Fruit ripening is a highly coordinated, genetically programmed, and irreversible process involving a series of physiological, biochemical, and organoleptic changes that lead to the development of a soft and edible ripe fruit with desirable quality attributes [1]. All biochemical and physiological changes during fruit ripening are driven by a cascade of molecular events, starting with the activation of signalling pathways [2]. These signals stimulate specific transcriptional regulators, which are responsible for the coordinated expression of the fruit ripening-related genes that directly control the biochemical processes [3,4].

The plant growth regulator ethylene is the major signalling molecule controlling most aspects of fruit ripening in climacteric fruits [2]. Ethylene response is regulated at multiple levels, from hormone synthesis and perception to signal transduction and transcriptional regulation [5]. In climacteric fruits, ethylene stimulates its own biosynthesis in an autocatalytic process [6], leading to the concept of two systems for ethylene production. System 1 and System 2 are characterized by its negative and positive feedback regulation by ethylene, respectively [2]. In persimmon, previous studies have observed that the emission of ethylene after harvesting is greater in immature fruits [7]. Nevertheless, the persimmon is classified as climacteric because it produces a small but significant amount of ethylene during ripening and is sensitive to exogenously applied ethylene [8].

The pathway for ethylene biosynthesis has been elucidated in apple and other fruits such as the avocado, banana, tomato, and Chinese jujube [9,10,11,12]. This process is led by two key biosynthetic enzymes: 1-aminocyclopropane-1-carboxylate (ACC) synthase (ACS), which converts S-adenosyl-L-methionine (SAM) to ACC, and ACC oxidase (ACO), which converts ACC to ethylene [13]. The pathway for ethylene signal transduction involves ethylene response factors (ERFs), ethylene insensitive-like factors (EILs), ethylene response sensors (ERSs), and ethylene receptors (ETRs), which also play a key role in persimmon fruit ripening [5,14,15]. ERFs are plant-specific transcription factors belonging to the large AP2/ERF superfamily and act as critical downstream components of the ethylene signalling pathway [16]. Numerous *ERF* genes are further induced by two downstream transcription factors, EIN3 and EIN3-LIKE1 (EIL1) [17]. Downregulation of ethylene responses by its receptors is released by binding to this phytohormone [18]. Consequently, the alteration of ethylene signalling and response components affects fruit ripening [19]. Understanding the mechanisms underlying the specificity of ethylene action requires unravelling the components mediating ethylene responses that are specific to each step of fruit ripening [20]. The available literature on the ethylene effect on persimmon ripening focuses on postharvest traits, shelf life, and extended cold storage [21,22], whereas the mechanisms of transcriptional regulation of ethylene biosynthesis and signal transduction during persimmon fruit ripening on the tree remain poorly known.

In the present study, we analysed the expression of 13 genes involved in ethylene biosynthesis and response pathways, colour change, and fruit softening along the natural persimmon ripening process. Samples from fruits of seven accessions were used to profile the expression of *DkACO1*, *DkACO2*, *DkACS2*, *DkEIL1*, *DkEIL3*, *DkERF8*, *DkERF16*, *DkERF18*, *DkERS1*, *DkETR2*, *DkETR3*, *DkPSY,* and *DkXTH9* from an early stage of fruit development to overripening. Some of these genes were already described by the literature as likely candidates to initiate and regulate the ethylene response and deastringency removal in persimmon fruit under postharvest treatments. Our purpose was to characterize their specific accumulation during on-tree fruit maturation. A principal component analysis (PCA) showed differences in the temporal expression of genes and correlations with specific phenotypic fruit traits. This information is of great interest for unravelling the ethylene-involved molecular mechanisms and provides a description of the gene network involved in persimmon fruit ripening on the tree.

## 2. Results

### 2.1. Persimmon Ripening Physiological Parameters

**Figure 1:**
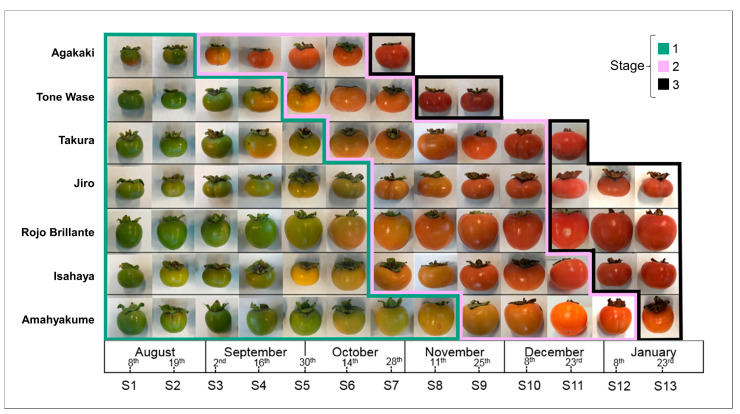
Fruit development and ripening observed in seven accessions across sampling dates. Stage one: samples prior to commercial ripening (**cyan**). Stage two: samples collected between commercial and physiological ripening (**pink**). Stage three: samples collected after physiological ripening (**black**).

Seven persimmon accessions with contrasting fruit maturation dates and different genetic backgrounds were selected from the Instituto Valenciano de Investigaciones Agrarias (IVIA) germplasm collection [23]. The number of samplings for each accession depended on the time required to reach its physiological maturation. The accession ‘Agakaki’ showed the fastest rate of fruit development and ripening, requiring 7 samplings for full ripening. On the other hand, ‘Jiro’, ‘Rojo Brillante’, ‘Isahaya’, and ‘Amahyakume’ needed 13 fruit samplings to cover the entire process of fruit development and ripening (Figure 1).

**Figure 2:**
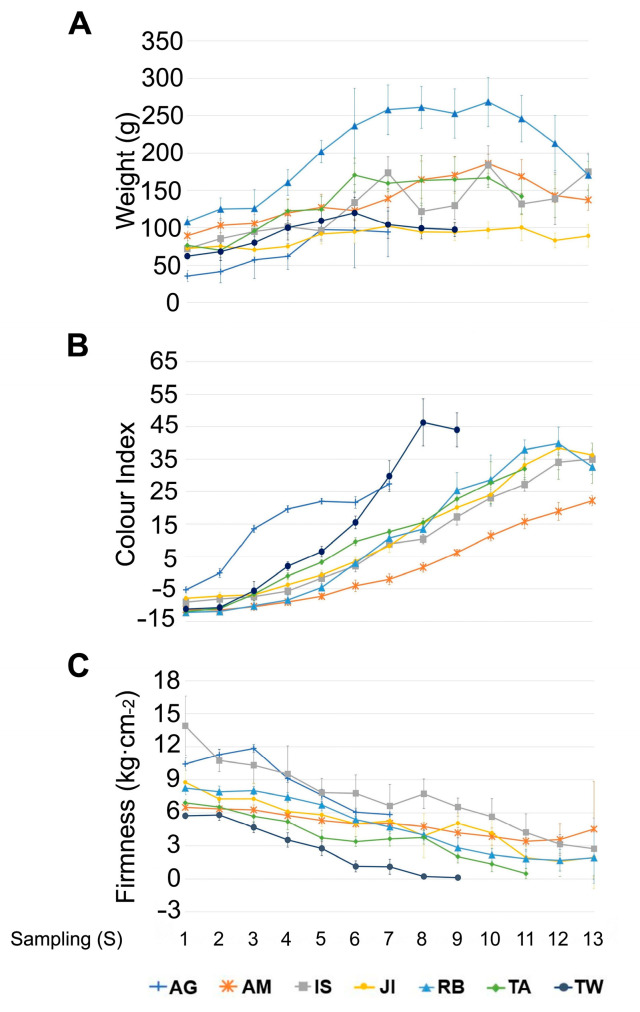
Phenotypical traits across fruit development in seven persimmon accessions. (**A**) Fruit weight, (**B**) fruit colour index, and (**C**) fruit firmness. AG: Agakaki, AM: Amahyakume, IS: Isahaya, JI: Jiro, RB: Rojo Brillante, TA: Takura, TW: Tone Wase. Standard deviations (SD) are represented in vertical error bars.

Accessions in the study increased their fruit weight during fruit development progression (Figure 2A). At the commercial maturity stage, all accessions reached 90% of their maximum fruit weight, except ‘Agakaki’, which at that stage achieved 50% of its maximum weight. In every accession, a slight decrease in weight was observed from its maximum value until reaching full physiological maturity. Among the different accessions, ‘Rojo Brillante’ reached the highest fruit weight average, whereas ‘Agakaki’, ‘Jiro’, and ‘Tone Wase’ showed the lowest fruit weight, respectively.

Regarding skin colour, the colour index (CI) shifted from negative (green skin) to positive values (red skin) as fruit development progressed (Figure 2B). ‘Agakaki’ and ‘Tone Wase’ showed a faster colour change compared to the other accessions, reaching their highest CI value on 7th and 8th sampling dates, respectively. These two accessions were the fastest to complete their fruit ripening cycle. The rest of the accessions reached their highest CI value on the 12th or 13th sampling dates. The highest CI value was observed in ‘Tone Wase’ and ‘Amahyakume’ was the accession showing the lowest CI level at the end of the experiment. ‘Isahaya’, ‘Jiro’, ‘Rojo Brillante’, and ‘Takura’ shared a similar colour evolution pattern. Parameters shown in Figure 2 have been fit to a polynomial function (Figure S1), and a non-parametric statistical test has been performed (Table S1).

Commercial and physiological maturity dates were estimated considering CI threshold values of five and 30, respectively. They were similar to those provided by the IVIA germplasm bank database (Table 1). These experimental commercial and physiological dates were employed to limit three fruit developmental stages for the different accessions, except for stage three in ‘Agakaki’ and ‘Amahyakume’. For these two accessions, the last sampling point was considered as stage three despite the fact that the CI was under 30 (Figure 1). After the last sampling, fruits fell naturally from the tree, indicating that they were overripe. Fruit development stage one showed the greatest variability. Accessions needed between two and eight samplings before reaching commercial maturity. This represents a difference of 90 days between the earliest accession (‘Agakaki’) and the latest one (‘Amahyakume’). Stage two, which ranges from commercial to physiological maturity, showed lower variability. In this case, the number of samplings fluctuated between 3 and 5. Finally, stage 3 (overripe fruit) varied between one and three samplings. ‘Rojo Brillante’ and ‘Jiro’ showed the greatest ability to maintain overripe fruit attached to the tree.

Fruit softening showed large differences among accessions. The initial firmness ranged from 5.7 kg.cm^−2^ to 13.9 kg.cm^−2^ in ‘Tone Wase’ and ‘Isahaya’, respectively. Accessions showing lower firmness values were ‘Takura’ and ‘Tone Wase’, which lost 97% and 98% of their initial firmness, respectively. In contrast, ‘Agakaki’ and ‘Amahyakume’ were the accessions with lower firmness loss. The rest of them lost around 80% of the initial value at the physiological ripening stage (Figure 2C). In order to describe mathematically how these three phenotypic traits behave throughout sampling, we have performed a least-squares regression analysis and we have obtained functions describing a 2nd order polynomial curve fit for each accession (Figure S1).

### 2.2. Expression Analysis of the Ethylene Pathway and Ripening Related Genes

**Figure 3:**
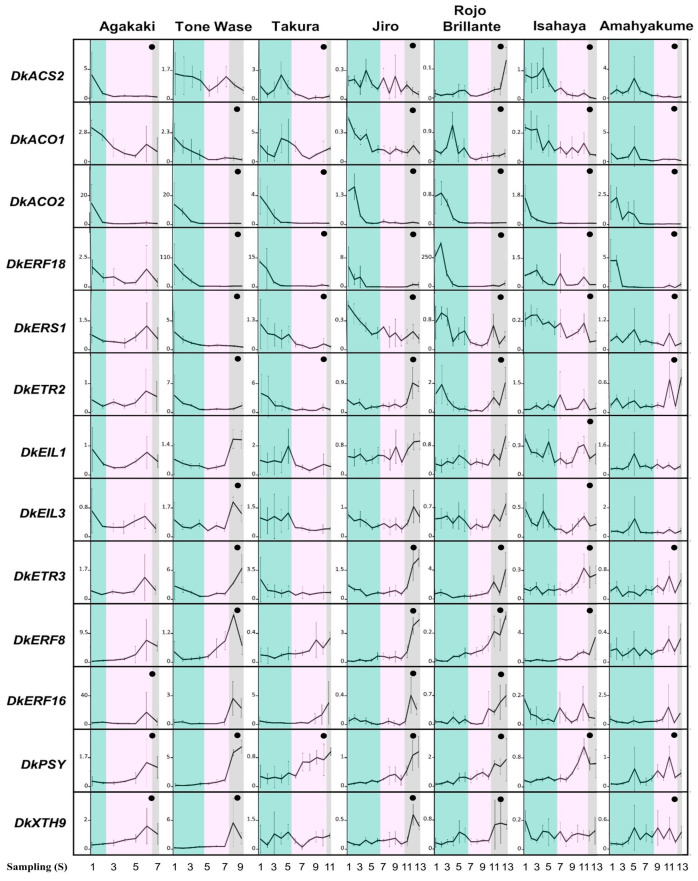
Relative expression analysis of ripening and ethylene-pathway-related genes by real-time quantitative PCR during fruit development in seven accessions. Stage one: samples prior to commercial ripening (**cyan**). Stage two: samples collected between commercial and physiological ripening (**pink**). Stage three: samples collected after physiological ripening (**black**). (•) Statistically significant difference (*p*-value < 0.05). Standard deviations (SD) are represented in vertical error bars.

The expression of thirteen persimmon genes related to ethylene synthesis and response and fruit ripening (Table 2) were analysed in all accessions during fruit development (Figure 3). Relative expression data after qRT-PCR showed statistically significant differences during development (Table S1).

Genes were classified into three groups according to their expression pattern. The first group included genes showing a decreasing pattern as the fruit ripens, reaching their highest expression levels at stage one. This set comprises genes encoding enzymes involved in ethylene biosynthesis (*DkACS2*, *DkACO1,* and *DkACO2*) as well as regulators of ethylene biosynthesis (*DkERF18*) and ethylene response sensors (*DkERS1*). The expression profile of *DkACS2* was similar in all accessions except for ‘Rojo Brillante’, which led to a sharp upregulation in S12. It should be noted that the *DkERF18* maximum expression level varied between 1.5 and 8.0 in most accessions, but it reached exceptionally high values around 270 and 100 in ‘Rojo Brillante’ and ‘Tone Wase’ samples, respectively.

A second group contained genes with an increasing expression pattern. This set includes genes encoding ethylene receptors (*DkERF8* and *DkERF16*), genes associated with colour and firmness changes (*DkPSY* and *DkXTH9*), and genes involved in ethylene reception and response regulation (*DkETR3*). The expression profile of these genes was similar in most accessions except for *DkETR3* and *DkXTH9* in ‘Takura’ and *DkERF16* in ‘Isahaya’. *DkERF16* expression level oscillated between values from 0.2 to 5, but it reached a maximum value of 17 in ‘Agakaki’. Finally, a third group was composed of genes with an accession-dependent pattern of expression: *DkEIL1*, *DkEIL3* (ethylene insensitive 3-like protein family), and *DkETR2* (ethylene receptor).

### 2.3. Association between Gene Expression and Phenotypic Data

**Figure 4:**
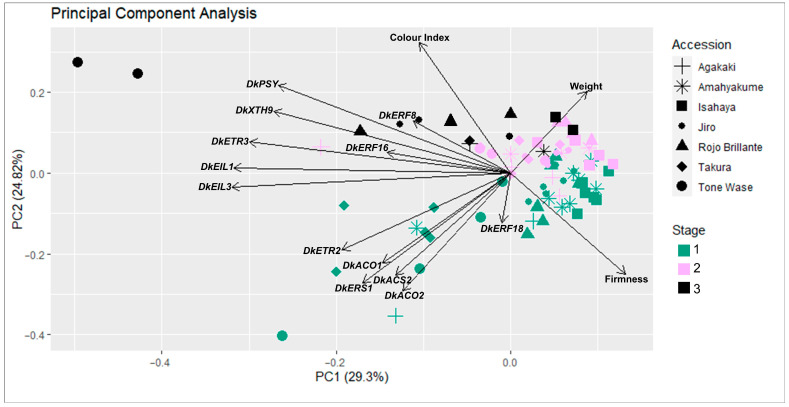
Phenotypic and genotypic expression data representation of each accession across fruit development in a two-dimensional space defined by the first two principal components. Stage one: samples prior to commercial ripening (**cyan**). Stage two: samples collected between commercial and physiological ripening (**pink**). Stage three: samples collected after physiological ripening (**black**).

A PCA was conducted in order to check the distribution of the samples. The first three principal components (PCs) accounted for 64.7% of the total variability observed in the dataset. To visualize the distribution of all the accessions, a scatter plot was created in the two-dimensional space defined by the first two PCs (Figure 4). The spatial distribution of samples revealed a grouping according to the development stage. Overall, samples belonging to stage one were distributed ranging from −0.2 to 0.05 PC2 values. Stage two and stage three samples were plotted between 0.0–0.1 and >0.1 values of PC2, respectively. Three off-type samples belonging to ‘Tone Wase’ S1, S2, and S9 were observed.

In this analysis, genes were distributed into two clearly differentiated groups. The first one included *DkACO1*, *DkACO2*, *DkACS2*, *DkERF18*, *DkERS1,* and *DkETR2*. As observed, they were correlated with stage one samples. Among these variables, *DkACO2* and *DkERS1* presented the higher component loading. On the other side, a second group of genes (*DkEIL1*, *DkEIL3 DkERF8*, *DkERF16*, *DkETR3*, *DkPSY,* and *DkXTH9*) correlated well with samples belonging to developmental stages two and three. *DkEIL1* and *DkEIL3* loading components were represented in the intermediate region between the groups described above, in agreement with the transcriptomic analysis. The highest component loadings were in *DkEIL1*, *DkEIL3,* and *DkETR3*.

Regarding phenotypic traits, CI and firmness were inversely correlated, with fruit weight perpendicular to them. Furthermore, we observed a direct relationship between the first group of genes and fruit weight, since genes related to ethylene biosynthesis became downregulated from stage two, when fruit increases in weight. The relationship between *DkPSY* and *DkXTH9* genes and CI and firmness variables also indicates their possible involvement in fruit softening and colour change.

## 3. Discussion

### 3.1. A Putative Role of DkACO1, DkACO2, and DkACS2 Genes in the System 1 to System 2 Transition

The climacteric fruit ripening process is regulated by ethylene biosynthesis, showing the auto inhibitory System 1 at early stages of fruit development and lately the autocatalytic System 2, in concordance with fruit ripening [24]. Unlike typical climacteric fruit, persimmon fruit has some unique characteristics: (1) ethylene biosynthesis is only induced when the fruit is detached from the tree; and (2) the amount of ethylene produced is higher in early stages of fruit development [7,25]. In addition, ethylene concentration in persimmon fruit is lower and more similar to non-climacteric levels [14], and the measurement of ethylene produced by attached fruits is extremely difficult without interfering with its natural biosynthesis cycle [26]. Furthermore, there is strong evidence that *ACO* gene expression correlates positively with ethylene production rates [27], which makes the expression of this gene a close marker of ethylene production across fruit development. Thus, our data suggest that the ethylene production rate is higher in stage one. Coding genes for key enzymes in ethylene biosynthesis (*DkACS2*, *DkACO1*, and *DkACO2*) showed a higher expression level in stage 1 samples and a decreasing trend towards stages two and three, in complete agreement with previous observations of ethylene synthesis in immature persimmon fruit. ACS was initially considered the rate-limiting enzyme in ethylene production. However, recent studies have demonstrated that *ACO* plays a crucial role in regulating ethylene production across multiple processes, including the ripening process [28]. The molecular mechanisms of the autocatalytic System 2 were identified by studying *ACO* and *ACS* gene expression during the transition from System 1 to System 2 in ripening tomatoes [2]. Previous works suggest that in tomatoes, System 1 is involved in negatively regulating *LeACS1A* and *LeACS6* genes. Furthermore, System 2 is activated due to the up-regulation of *LeACS2* and *LeACS4* genes, which is facilitated by positive feedback from ethylene [24,29]. In our study, the *DkACO2* expression level decreased drastically to values close to zero before finishing stage one, which could be interpreted as a System 1 auto-inhibitory behaviour. Additionally, *DkACO1* and *DkACS2* remained active but were down-expressed in stages two and three. They increase their expression under stress conditions such as wounding [30], water stress [31], or detached fruits [32], which could sustain a putative autocatalytic production of ethylene (System 2). Therefore, the expression of *DkACO1*, *DkACO2,* and *DkACS2* genes explain the previously reported particularities of ethylene production during fruit development in persimmon.

### 3.2. Ethylene Response Genes Are Differentially Regulated during Fruit Development

Ethylene is perceived by a family of transmembrane proteins located in the endoplasmic reticulum that play an important role in fruit ripening regulation. These ETRs are the first element of the signalling cascade triggered by ethylene binding [14,33]. In our study, *DkETR2* showed accession-dependent peaks of expression in stages one and 3, suggesting its participation in early and late events of fruit development. In turn, *DkETR3* showed an increasing expression trend from stage two to stage three, except for ‘Takura’. These results suggest that *DkETR3* expression correlates with the fruit ripening process, as previously described in tomato where it seems to be crucial to ripen [20,34]. However, the physiological significance of the increase in expression of receptor genes during fruit ripening is not so clear given their negative regulation role in ethylene signal transmission. In our study, *DkERS1* expression was higher at stage one, supporting its role in early ethylene response. In close agreement with our data, a higher expression of *DkERS1* in immature fruits has been postulated to sense the secondary ethylene caused by the autocatalytic ethylene production in detached fruit [14]. In another work, the exogenous application of ethylene produced rapid fruit softening preceded by the induction of *DkERS1* expression [13]. A small amount of ethylene could be enough to switch off the inhibitory effect of ethylene receptors and then trigger ripening-related responses [8]. Finally, as the last components of the ethylene signal pathway and direct mediators regulating ethylene-responsive genes, ERFs have been shown to be involved in various aspects of ripening including ethylene biosynthesis, colour change, and fruit softening [35]. The APETALA2/ethylene response factor (AP2/ERF) superfamily is defined by the presence of the AP2/ERF domain [36]. ERF subfamily members have been shown to bind the GCC-box (AGCCGCC) found in ethylene-responsive genes, whereas the DREBs subfamily usually binds to the DRE cis-acting element (A/GCCGAC) [35]. *DkERF18* showed an expression profile similar to *DkACO1*, *DkACO2,* and *DkACS1* genes. This correlation has been reported previously in persimmon where the involvement of *DkERF18* in the autocatalytic ethylene system occurs through activation of the *DkACS2* gene via the ACCGAC motif [37]. Therefore, our results may indicate a feedback regulation between *DkERF18* and ethylene production.

### 3.3. Genes Leading to Morphological Changes

Persimmon fruit shows a double sigmoidal growth curve with three phases, two phases of rapid growth (phase I and III), separated by a slow growth period (phase II). The phase II coincides with the warm period and a prolonged summer often delays the onset of the final expansion in phase III [38]. In our study, fruit weight increased from the first sampling until reaching its maximum value in stage two, but it did not correlate with the expression of any studied gene.

External colour change is the most evident change during persimmon fruit development. Thus, skin colour is used as the most common non-destructive harvest index for persimmons. Most persimmon cultivars are considered ready for harvest when they show a complete orange to orange-red colour with no visible green background [39,40]. This process is led by chlorophyll degradation, which uncovers previously synthesized pigments, usually carotenoids [41,42,43]. Several studies report phytoene synthase, encoded by *DkPSY*, as the key role enzyme in carotenoid biosynthesis in persimmon [44,45]. *DkPSY* gene expression fits well with these findings, since it is higher in stage two and three, achieving its maximum level at stage three. It is well known that *ERF* genes regulate *PSY* gene expression by directly interacting with the ATCTA motif [35,46,47,48]. The highly coincident expression patterns of *DkPSY*, *DkERF8,* and *DkERF16* support the involvement of these factors in colour change processes, which could also be dependent on the activation of other genes, such as *DkEIL1* and *DkEIL3*. Indeed, *DkEIL1* has been proposed to play an important role in the colour change process under ethylene and 1-methylcyclopropene treatments during fruit storage [5].

Fruit texture is generally considered as a quantitative trait, which is regulated by multiple genes such as polygalacturonase, xyloglucan endo-transglucosylase/hydrolases, β-galactosidase, and pectate lyase. Cell-wall-related genes, such as *DkXTH9*, were found to be associated with fruit firmness. In our study, *DkXTH9* expression increased in stage three concomitantly with *DkERF8* and *DkERF16* genes, as described for *DkPSY*. The proliferation of excellent molecular studies addressing the molecular regulation of deastringency mechanisms by hypoxia treatments in persimmon fruit under postharvest conditions has enlightened the fruit ripening field. Under this postharvest model, both *DkERF8* and *DkERF16* genes trans-activate the *DkXTH9* promoter and other genes involved in cell wall dynamics measured by dual-luciferase assay [15,37]. These results support the participation of *DkERF8* and *DkERF16* in fruit softening during natural persimmon fruit ripening, as suggested by our gene expression correlation study.

In our opinion, this study contributes to describing at the molecular level the intriguing production of ethylene in young fruits of persimmon when detached from the tree, sustained by the high expression of the ethylene biosynthetic genes *DkACS2*, *DkACO1*, and *DkACO2* and the *DkERF18* factor in early stages of fruit development. In addition, the coordinated expression of the regulatory genes *DkERF8* and *DkERF16* and the effector genes *DkPSY* and *DkXTH9* suggests a role of these factors in fruit skin colour change and softening processes, to be confirmed in subsequent studies. A better understanding of these processes will provide valuable information for breeding new varieties, optimizing postharvest strategies for persimmon.

## 4. Materials and Methods

### 4.1. Plant Material

Fruit samples from seven persimmon accessions were used. Trees were grown under standard agricultural practices in an orchard located at IVIA facilities (39.585346, −0.395117). These accessions were chosen to represent different maturity dates and genetic backgrounds according to previous work (Table 1) [23]. In each sampling, six fruits with similar shape, colour, and size were collected from each accession every two weeks from the 8th of August until physiological ripening.

### 4.2. Phenotypic Evaluation and Sample Collection

Flesh firmness, colour index (CI), and fruit weight were measured at each sampling date. Flesh firmness was determined as the maximum force applied to the fruit flesh until tissue breaks using a Shimadzu (Kyoto, Japan) EZ-L penetrometer equipped with a one-cm2 probe. Fruit skin colour was determined as the mean of three measurements per fruit using a Chroma Meter Minolta (Osaka, Japan) CR-300 with a DP-301 data processor. Colour determinations were made as 2° observer and Standard illuminant C calibration. Data are expressed as Hunter Lab coordinates. Colour index was determined as (CI = (1000 ×a)/(L×b)) [49]. Commercial and physiological ripening stages were determined by the CI. Commercial maturity was reached when the peel colour changed from green to orange, corresponding to a CI > 5. Physiological maturity was established when the CI > 20. It must also be fulfilled that with respect to the previous sampling there exists a difference of five points between them. For clarity purposes, we have divided fruit development into three stages according to these CI-based commercial and physiological maturity dates: stage one when CI < 5; stage two when 5 < CI < 30; and finally stage three for CI > 30. For every sampling date, flesh from two fruits was grouped as a biological replicate in each accession. Samples were frozen with liquid nitrogen, finely ground into powder, and stored at −80 °C until RNA extraction.

### 4.3. RNA Extraction and Gene Expression

RNA extraction from 70 mg fruit samples was made using the Plant/Fungi Total RNA Purification Kit (Norgen Biotek Corp., Thorold, ON, Canada), modified with the addition of 2% PVP and 2% β-mercaptoethanol to the lysis buffer. Purified RNA was quantified by Qubit (Invitrogen, Carlsbad, CA, USA) fluorometry and integrity was checked by agarose electrophoresis. RNA was reverse transcribed to cDNA in a total volume of 10 μL, using the PrimeScript RT Reagent Kit (Takara Bio, Otsu, Japan).

The qRT-PCR was performed on a StepOnePlus Real-Time PCR System (LIFE TECHNOLOGIES, Carlsbad, CA, USA), using 1 µL of 10X diluted cDNA, SYBR premix Ex Taq (Tli RNaseH plus) (TAKARA BIO, Kusatsu, Japan) [50]. *DkTUA* was used as a housekeeping gene reference [51]. Gene-specific primers used for expression analysis are listed in Table S2. Primers were designed using coding sequences from NCBI genes and Primer3 software v. 4.1.0 (https://primer3.org) accessed on 10 November 2020. The specificity of the designed primers was checked by the presence of a single peak in the dissociation curve after the amplification.

### 4.4. Data Analysis

The relative standard curve method was employed to measure the relative expression and generate the raw gene expression matrix. Data analysis was carried out using RStudio integrated development environment for R 2022.02.2 (https://rstudio.com/) accessed 20 November 2020 and Bioconductor framework 3.16 (www.bioconductor.org) accessed 5 May 2023. Morphological trait measurements and gene expression levels were analysed by the non-parametric statistical test Kruskal–Wallis using the IBM SPSS Statistics v.29.0.1.0 software. In addition, a pairwise comparison was performed to separate differences; the level of significance was *p* < 0.05. The ggfortify R package 0.4.11 [52] was used to perform a PCA [53] for representing a two-dimensional projection of normalized gene expression and phenotypic data correlations. Least-squares regression analysis and 2nd order polynomial curve fitting from Figure S1 were performed using Microsoft Excel 365 software [54].

## Figures and Tables

**Table 1:** Commercial and physiological ripening dates of selected accessions.

Accession	Commercial Ripening *	Physiological Ripening *
Agakaki	5th September	8th November
Amahyakume	29th November	25th January
Isahaya	12th November	28th January
Jiro	27th October	5th January
Rojo Brillante	29th October	4th January
Takura	19th October	7th January
Tone Wase	23rd September	8th November

* Ripening dates are the average of four years. Data obtained from the IVIA persimmon germplasm bank database.

**Table 2:** List of the 13 analysed genes related to persimmon ripening.

Gene Name	Accession Number	Protein Name	Function
*DkACO1*	AB073008	1-aminocyclopropane-1-carboxylate oxidase 1.	Key enzyme in ethylene biosynthesis.
*DkACO2*	AB073009	1-aminocyclopropane-1-carboxylate oxidase 2.	Key enzyme in ethylene biosynthesis.
*DkACS2*	AB073006	1-aminocyclopropane-1-carboxylate synthase.	Key enzyme in ethylene biosynthesis.
*DkEIL1*	JN256070	Ethylene insensitive 3-like protein EIL1.	Ethylene signalling pathway.
*DkEIL3*	XM_052331725 (*D. lotus*)	Ethylene insensitive 3-like 3 protein EIL1.	Ethylene signalling pathway.
*DkERF8*	JN256078	Ethylene response factor 8.	Regulator of ethylene biosynthesis. Possible promoter of fruit ripening by cell wall modification.
*DkERF16*	KJ170916	Ethylene response factor 16.	Regulator of ethylene biosynthesis. Possible promoter of fruit ripening by cell wall modification.
*DkERF18*	KJ170918	Ethylene response factor 18.	Regulator of ethylene biosynthesis.
*DkERS1*	AB164038	Ethylene response sensor 1.	Ethylene perception and regulators of the ethylene-response pathway.
*DkETR2*	AB243790	Ethylene receptor ETR2.	Ethylene perception and regulators of the ethylene-response pathway.
*DkETR3*	KX871217	Ethylene receptor ETR3.	Ethylene perception and regulators of the ethylene-response pathway.
*DkPSY*	FJ713744	Phytoene synthase.	Catalysis in the first committed step of the carotenoid biosynthesis pathway.
*DkXTH9*	KF318889	Xyloglucan endotransglucosylase/hydrolase 9.	Cell wall construction of growing tissues.

## Data Availability

Data recorded in the current study are available in all tables and figures of the manuscript.

## Supplementary Materials

The following supporting information can be downloaded at: https://www.mdpi.com/article/10.3390/plants12162895/s1, Figure S1. Least-squares regression analysis and 2nd order polynomial curve fit for each phenotypic trait; Table S1. Results of non-parametric statistical test Kruskal–Wallis analysis of relative gene expression level and morphological traits in studied accessions; Table S2. List of gene-specific primers used for real-time quantitative PCR expression analysis.
