# Supplementary material for: Physiological Changes and Transcriptomic Analysis throughout On-Tree Fruit Ripening Process in Persimmon (Diospyros kaki L.)
Source: Plants (Basel). 2023 Aug 8;12(16):2895. doi: 10.3390/plants12162895 (PMC10457761; doi:10.3390/plants12162895)
Supplement: Supplementary file 1 [file plants-12-02895-s001.zip › plants-2508299-supplementary.pdf]

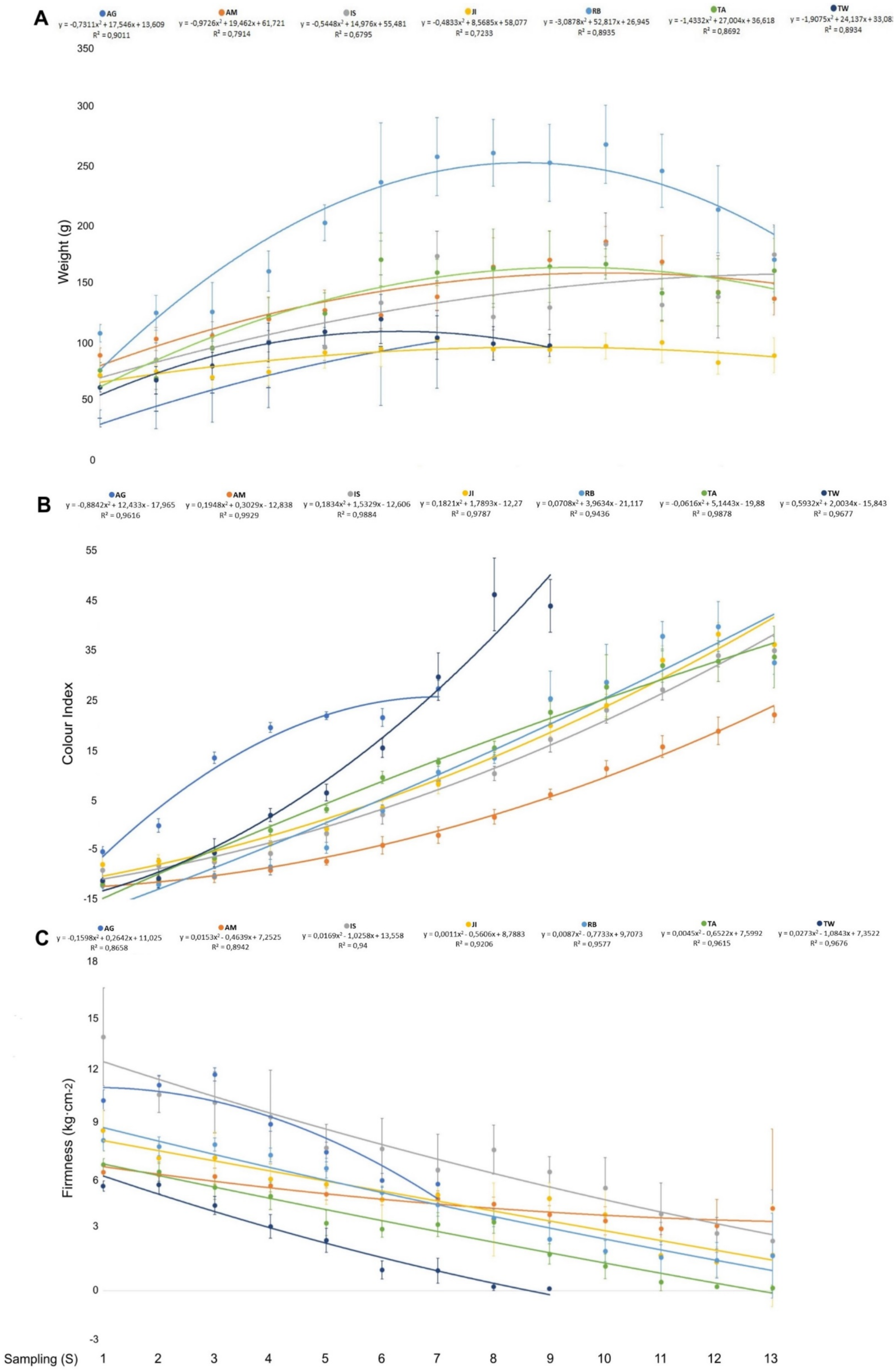

Figure S1. Least-squares regression analysis and 2nd order polynomial curve fit for each phenotypic trait. (A) Weight, (B) Colour Index and (C) Firmness. AG: Agakaki, AM: Amahyakume, IS: Isahaya, JI: Jiro, RB: Rojo Brillante, TA: Takura, TW: Tone Wase. Standard deviations (SD) are represented in vertical error bars.

Table S1. Results of non-parametric statistical test Kruskal-Wallis analysis of relative gene expression level and morphological traits in studied accessions. Significant values are highlighted in yellow ($p<0.05$). Comp.=Comparisons, Sig.=Significance.

AGAKAKI

Test: Kruskal-Wallis (p<0.05)

TRAIT / GENE	Sig.
WEIGHT	0,000
CI	0,000
FIRNESS	0,000
DkERF16	0,395
DkERF8	0,026
DkERF18	0,228
DkACO1	0,143
DkACO2	0,076
DkACS2	0,038
DkERS1	0,417
DkETR3	0,167
DkEIL1	0,052
DkEIL3	0,261
DkPSY	0,041
DkXTH9	0,036
DkETR2	0,391

Pairwise Comparisons (p<0.05)

WEIGHT		CI		FIRMNESS	
Comp.	Sig.	Comp.	Sig.	Comp.	Sig.
S1-S5	0,000	S1-S7	0,000	S7-S3	0,000
S1-S7	0,000	S2-S7	0,000	S6-S3	0,000
S1-S6	0,000	S1-S5	0,000	S7-S2	0,001
S2-S5	0,000	S1-S6	0,001	S6-S2	0,001
S2-S7	0,000	S3-S7	0,001	S5-S3	0,004
S2-S6	0,001	S2-S5	0,002	S7-S1	0,005
S3-S5	0,018	S2-S6	0,003	S6-S1	0,006
S1-S4	0,020	S1-S4	0,004	S5-S2	0,011
S3-S7	0,023	S2-S4	0,014	S7-S4	0,047
S3-S6	0,028	S4-S7	0,044	S6-S4	0,053
S4-S5	0,055	S3-S5	0,076	S5-S1	0,057
S1-S3	0,063	S3-S6	0,086	S4-S3	0,084
S4-S7	0,067	S1-S3	0,098	S4-S2	0,167
S4-S6	0,082	S6-S7	0,141	S5-S4	0,266
S2-S4	0,099	S5-S7	0,157	S1-S3	0,296
S2-S3	0,237	S2-S3	0,216	S7-S5	0,381
S1-S2	0,479	S3-S4	0,238	S6-S5	0,412
S3-S4	0,653	S4-S5	0,551	S4-S1	0,452
S6-S5	0,857	S4-S6	0,590	S1-S2	0,512
S6-S7	0,928	S1-S2	0,662	S2-S3	0,678
S7-S5	0,928	S6-S5	0,955	S7-S6	0,955

DkERF16		*DkERF8*		*DkERF18*		*DkACO1*		*DkACO2*	
Comp.	Sig.	Comp.	Sig.	Comp.	Sig.	Comp.	Sig.	Comp.	Sig.
S05-S02	0,038	S01-S07	0,005	S04-S01	0,031	S05-S01	0,017	S04-S01	0,006
S07-S02	0,053	S01-S06	0,006	S07-S01	0,036	S07-S01	0,024	S04-S02	0,023
S04-S02	0,084	S02-S07	0,023	S05-S01	0,048	S05-S02	0,045	S06-S01	0,024
S03-S02	0,214	S02-S06	0,027	S04-S02	0,129	S04-S01	0,060	S07-S01	0,028
S05-S01	0,241	S03-S07	0,038	S07-S02	0,147	S07-S02	0,062	S06-S02	0,084
S07-S01	0,294	S03-S06	0,045	S04-S03	0,190	S04-S02	0,147	S03-S01	0,090
S05-S06	0,301	S01-S05	0,052	S05-S02	0,190	S03-S01	0,165	S07-S02	0,098
S06-S02	0,301	S01-S04	0,060	S07-S03	0,214	S06-S01	0,165	S05-S01	0,130
S07-S06	0,370	S02-S05	0,190	S06-S01	0,217	S05-S03	0,270	S04-S05	0,168
S04-S01	0,388	S02-S04	0,214	S05-S03	0,270	S05-S06	0,270	S04-S03	0,241
S05-S03	0,408	S03-S05	0,270	S04-S06	0,301	S07-S03	0,334	S03-S02	0,270
S07-S03	0,490	S03-S04	0,301	S03-S01	0,323	S07-S06	0,334	S05-S02	0,370
S04-S06	0,490	S04-S07	0,301	S07-S06	0,334	S03-S02	0,370	S06-S05	0,408
S01-S02	0,497	S04-S06	0,334	S05-S06	0,408	S06-S02	0,370	S07-S05	0,448
S04-S03	0,629	S05-S07	0,334	S02-S01	0,422	S02-S01	0,558	S02-S01	0,478
S03-S01	0,666	S01-S03	0,339	S06-S02	0,629	S05-S04	0,581	S04-S07	0,535
S05-S04	0,730	S05-S06	0,370	S06-S03	0,783	S04-S03	0,581	S06-S03	0,535
S06-S01	0,805	S01-S02	0,440	S04-S05	0,836	S04-S06	0,581	S04-S06	0,581
S07-S04	0,836	S02-S03	0,836	S03-S02	0,836	S07-S04	0,679	S07-S03	0,581
S03-S06	0,836	S04-S05	0,945	S07-S05	0,890	S05-S07	0,890	S03-S05	0,836
S05-S07	0,890	S06-S07	0,945	S04-S07	0,945	S03-S06	1,000	S06-S07	0,945

DkACS2		*DkERS1*		*DkETR3*		*DkEIL1*	
Comp.	Sig.	Comp.	Sig.	Comp.	Sig.	Comp.	Sig.
S07-S01	0,003	S03-S05	0,098	S02-S06	0,006	S03-S06	0,011
S07-S02	0,006	S03-S06	0,129	S02-S07	0,032	S03-S05	0,013
S03-S01	0,024	S03-S01	0,147	S02-S05	0,073	S04-S06	0,027
S03-S02	0,045	S04-S05	0,147	S04-S06	0,084	S04-S05	0,032
S06-S01	0,060	S07-S05	0,168	S02-S03	0,098	S03-S01	0,033
S04-S01	0,069	S04-S06	0,190	S02-S01	0,195	S04-S01	0,069
S05-S01	0,079	S04-S01	0,206	S01-S06	0,241	S03-S07	0,073
S06-S02	0,112	S07-S06	0,214	S04-S07	0,270	S03-S02	0,112
S04-S02	0,129	S07-S01	0,229	S03-S06	0,270	S04-S07	0,147
S05-S02	0,147	S02-S05	0,270	S02-S04	0,301	S04-S02	0,214
S07-S05	0,190	S02-S06	0,334	S05-S06	0,334	S02-S06	0,334
S07-S04	0,214	S02-S01	0,339	S04-S05	0,448	S02-S05	0,370
S07-S06	0,241	S03-S02	0,581	S04-S03	0,535	S07-S06	0,448
S07-S03	0,448	S04-S02	0,730	S07-S06	0,535	S02-S01	0,478
S03-S05	0,581	S07-S02	0,783	S01-S07	0,537	S07-S05	0,490
S03-S04	0,629	S03-S07	0,783	S03-S07	0,629	S07-S01	0,600
S02-S01	0,643	S03-S04	0,836	S04-S01	0,711	S03-S04	0,730
S03-S06	0,679	S06-S05	0,890	S05-S07	0,730	S02-S07	0,836
S06-S05	0,890	S06-S01	0,926	S01-S05	0,758	S01-S06	0,877
S04-S05	0,945	S04-S07	0,945	S01-S03	0,853	S01-S05	0,926
S06-S04	0,945	S01-S05	0,975	S03-S05	0,890	S05-S06	0,945

DkEIL3		*DkPSY*		*DkXTH9*		*DkETR2*	
Comp.	Sig.	Comp.	Sig.	Comp.	Sig.	Comp.	Sig.
S07-S06	0,045	S02-S07	0,009	S01-S06	0,009	S04-S06	0,073
S03-S06	0,084	S03-S07	0,013	S02-S06	0,011	S04-S03	0,084
S04-S06	0,084	S02-S06	0,016	S03-S06	0,013	S02-S06	0,084
S07-S05	0,098	S03-S06	0,023	S01-S07	0,045	S02-S03	0,098
S02-S06	0,129	S01-S07	0,069	S02-S07	0,062	S04-S05	0,214
S07-S01	0,156	S04-S07	0,084	S01-S05	0,069	S04-S07	0,214
S03-S05	0,168	S01-S06	0,102	S03-S07	0,073	S02-S05	0,241
S04-S05	0,168	S02-S05	0,112	S02-S05	0,098	S02-S07	0,241
S02-S05	0,241	S04-S06	0,129	S03-S05	0,112	S04-S01	0,422
S03-S01	0,241	S03-S05	0,147	S01-S04	0,147	S01-S06	0,422
S04-S01	0,241	S05-S07	0,301	S04-S06	0,190	S02-S01	0,459
S02-S01	0,323	S02-S04	0,370	S02-S04	0,214	S01-S03	0,459
S07-S02	0,629	S01-S05	0,371	S03-S04	0,241	S05-S06	0,581
S01-S06	0,711	S05-S06	0,408	S05-S06	0,370	S07-S06	0,581
S05-S06	0,730	S03-S04	0,448	S07-S06	0,490	S05-S03	0,629
S07-S03	0,783	S04-S05	0,490	S04-S07	0,535	S07-S03	0,629
S07-S04	0,783	S02-S01	0,600	S04-S05	0,679	S01-S05	0,758
S03-S02	0,836	S03-S01	0,688	S01-S03	0,688	S01-S07	0,758
S04-S02	0,836	S01-S04	0,781	S01-S02	0,734	S03-S06	0,945
S01-S05	0,951	S06-S07	0,836	S05-S07	0,836	S04-S02	0,945
S03-S04	1,000	S02-S03	0,890	S02-S03	0,945	S05-S07	1,000

TONE WASE

Test: Kruskal-Wallis (p<0.05)

TRAIT / GENE	Sig.
WEIGHT	0,000
CI	0,000
FIRNESS	0,000
DkERF16	0,053
DkERF8	0,014
DkERF18	0,006
DkACO1	0,030
DkACO2	0,005
DkACS2	0,261
DkERS1	0,010
DkETR3	0,014
DkEIL1	0,059
DkEIL3	0,029
DkPSY	0,006
DkXTH9	0,006
DkETR2	0,018

Pairwise Comparisons (p<0.05)

WEIGHT		CI		FIRMNESS	
Comp.	Sig.	Comp.	Sig.	Comp.	Sig.
S1-S6	0,000	S1-S8	0,000	S9-S1	0,000
S1-S5	0,000	S1-S9	0,000	S9-S2	0,000
S2-S6	0,000	S2-S8	0,000	S8-S1	0,000
S2-S5	0,001	S2-S9	0,000	S8-S2	0,000
S1-S7	0,001	S1-S7	0,000	S9-S3	0,000
S1-S4	0,002	S2-S7	0,000	S7-S1	0,000
S1-S8	0,002	S3-S8	0,000	S6-S1	0,001
S1-S9	0,003	S3-S9	0,001	S7-S2	0,001
S2-S7	0,005	S1-S6	0,002	S8-S3	0,001
S3-S6	0,005	S2-S6	0,003	S6-S2	0,001
S2-S4	0,009	S4-S8	0,003	S9-S4	0,005
S2-S8	0,010	S4-S9	0,005	S8-S4	0,010
S2-S9	0,015	S3-S7	0,009	S9-S5	0,015
S3-S5	0,019	S1-S5	0,013	S7-S3	0,019
S3-S7	0,074	S2-S5	0,019	S5-S1	0,021
S3-S4	0,111	S5-S8	0,022	S6-S3	0,022
S3-S8	0,119	S5-S9	0,032	S5-S2	0,026
S1-S3	0,136	S4-S7	0,052	S8-S5	0,028
S3-S9	0,152	S3-S6	0,054	S4-S1	0,056
S9-S6	0,173	S1-S4	0,067	S4-S2	0,067
S8-S6	0,215	S2-S4	0,094	S7-S4	0,124
S4-S6	0,229	S6-S8	0,100	S6-S4	0,142
S7-S6	0,313	S6-S9	0,132	S9-S6	0,176
S2-S3	0,340	S5-S7	0,190	S9-S7	0,199
S9-S5	0,367	S3-S5	0,196	S5-S3	0,229
S8-S5	0,436	S4-S6	0,203	S7-S5	0,250
S4-S5	0,457	S1-S3	0,248	S8-S6	0,261
S1-S2	0,577	S2-S3	0,319	S6-S5	0,280
S7-S5	0,583	S7-S8	0,330	S3-S1	0,288
S5-S6	0,645	S7-S9	0,405	S8-S7	0,292
S9-S7	0,723	S6-S7	0,501	S3-S2	0,325
S8-S7	0,818	S3-S4	0,513	S4-S3	0,415
S4-S7	0,846	S4-S5	0,524	S5-S4	0,697
S9-S4	0,873	S5-S6	0,524	S9-S8	0,818
S9-S8	0,901	S1-S2	0,870	S2-S1	0,935
S8-S4	0,972	S9-S8	0,887	S7-S6	0,944

DkERF16		*DkERF8*		*DkERF18*		*DkACO1*		*DkACO2*	
Comp.	Sig.	Comp.	Sig.	Comp.	Sig.	Comp.	Sig.	Comp.	Sig.
S03-S09	0,003	S02-S08	0,003	S07-S01	0,002	S09-S01	0,006	S06-S02	0,001
S03-S08	0,005	S03-S08	0,006	S07-S02	0,002	S06-S01	0,006	S06-S01	0,002
S05-S09	0,037	S02-S07	0,009	S05-S01	0,009	S05-S01	0,008	S07-S02	0,003
S06-S09	0,037	S04-S08	0,011	S06-S01	0,009	S09-S02	0,019	S07-S01	0,006
S05-S08	0,042	S03-S07	0,019	S05-S02	0,009	S06-S02	0,022	S06-S03	0,014
S06-S08	0,042	S02-S09	0,025	S06-S02	0,009	S05-S02	0,025	S09-S02	0,014
S04-S09	0,043	S02-S06	0,029	S07-S03	0,014	S08-S01	0,042	S05-S02	0,019
S04-S08	0,048	S04-S07	0,033	S09-S01	0,033	S07-S01	0,078	S09-S01	0,022
S03-S02	0,048	S05-S08	0,038	S09-S02	0,033	S09-S03	0,098	S05-S01	0,029
S07-S09	0,070	S03-S09	0,048	S05-S03	0,048	S09-S04	0,098	S07-S03	0,033
S07-S08	0,073	S03-S06	0,055	S06-S03	0,048	S08-S02	0,100	S06-S04	0,098
S01-S09	0,078	S01-S08	0,059	S04-S01	0,088	S06-S04	0,109	S09-S03	0,098
S01-S08	0,081	S04-S09	0,078	S04-S02	0,088	S06-S03	0,109	S04-S02	0,109
S03-S01	0,220	S04-S06	0,088	S07-S08	0,100	S05-S03	0,122	S06-S08	0,121
S03-S07	0,240	S05-S07	0,109	S09-S03	0,135	S05-S04	0,122	S05-S03	0,122
S02-S08	0,283	S01-S07	0,165	S07-S04	0,165	S07-S02	0,182	S04-S01	0,150
S05-S02	0,286	S02-S01	0,220	S05-S08	0,223	S04-S01	0,262	S08-S02	0,174
S06-S02	0,286	S05-S09	0,220	S06-S08	0,223	S03-S01	0,262	S07-S04	0,182
S04-S02	0,311	S05-S06	0,240	S08-S01	0,262	S08-S04	0,305	S07-S08	0,206
S02-S09	0,311	S06-S08	0,305	S08-S02	0,262	S08-S03	0,305	S08-S01	0,223
S03-S04	0,337	S02-S05	0,311	S04-S03	0,286	S09-S07	0,311	S06-S05	0,364
S03-S05	0,364	S01-S09	0,311	S07-S09	0,337	S06-S07	0,337	S09-S04	0,393
S03-S06	0,364	S09-S08	0,328	S06-S04	0,364	S05-S07	0,364	S09-S08	0,403
S07-S02	0,423	S03-S01	0,337	S05-S04	0,364	S04-S02	0,488	S06-S09	0,423
S01-S02	0,455	S01-S06	0,337	S09-S08	0,431	S03-S02	0,488	S04-S03	0,423
S05-S01	0,749	S03-S05	0,455	S03-S02	0,522	S07-S03	0,522	S03-S02	0,423
S06-S01	0,749	S04-S01	0,455	S03-S01	0,522	S07-S04	0,522	S05-S04	0,455
S05-S07	0,790	S07-S08	0,519	S08-S03	0,583	S09-S08	0,650	S05-S08	0,459
S06-S07	0,790	S04-S05	0,594	S07-S05	0,631	S08-S07	0,650	S08-S03	0,519
S04-S01	0,790	S02-S04	0,631	S07-S06	0,631	S02-S01	0,669	S03-S01	0,522
S04-S07	0,831	S06-S07	0,669	S05-S09	0,631	S06-S08	0,685	S07-S05	0,557
S09-S08	0,867	S09-S07	0,709	S06-S09	0,631	S05-S08	0,720	S07-S09	0,631
S05-S04	0,957	S02-S03	0,790	S09-S04	0,669	S09-S05	0,915	S06-S07	0,749
S06-S04	0,957	S03-S04	0,831	S04-S08	0,685	S09-S06	0,957	S01-S02	0,873
S07-S01	0,957	S05-S01	0,831	S05-S06	1,000	S06-S05	0,957	S09-S05	0,915
S05-S06	1,000	S06-S09	0,957	S01-S02	1,000	S03-S04	1,000	S04-S08	0,943

DkACS2		*DkERS1*		*DkETR3*		*DkEIL1*	
Comp.	Sig.	Comp.	Sig.	Comp.	Sig.	Comp.	Sig.
S09-S03	0,025	S09-S02	0,001	S04-S09	0,001	S05-S09	0,002
S05-S03	0,037	S09-S01	0,002	S05-S09	0,001	S05-S08	0,005
S09-S07	0,055	S09-S03	0,003	S04-S08	0,011	S06-S09	0,016
S09-S01	0,062	S05-S02	0,022	S05-S08	0,011	S06-S08	0,030
S05-S07	0,078	S08-S02	0,023	S04-S02	0,033	S05-S02	0,062
S09-S02	0,088	S07-S02	0,033	S05-S02	0,033	S01-S09	0,062
S05-S01	0,088	S08-S01	0,048	S07-S09	0,043	S04-S09	0,070
S09-S04	0,098	S05-S01	0,048	S06-S09	0,055	S03-S09	0,078
S05-S02	0,122	S08-S03	0,059	S04-S01	0,070	S01-S08	0,090
S05-S04	0,135	S05-S03	0,062	S05-S01	0,070	S04-S08	0,100
S06-S03	0,182	S07-S01	0,070	S04-S03	0,070	S05-S07	0,109
S09-S08	0,242	S06-S02	0,070	S05-S03	0,070	S03-S08	0,110
S05-S08	0,305	S09-S04	0,078	S03-S09	0,135	S07-S09	0,135
S06-S07	0,311	S07-S03	0,088	S01-S09	0,135	S07-S08	0,174
S06-S01	0,337	S04-S02	0,109	S07-S08	0,159	S05-S03	0,182
S09-S06	0,364	S09-S06	0,122	S04-S06	0,165	S05-S04	0,200
S08-S03	0,403	S06-S01	0,135	S05-S06	0,165	S05-S01	0,220
S06-S02	0,423	S06-S03	0,165	S06-S08	0,189	S02-S09	0,220
S05-S06	0,455	S04-S01	0,200	S04-S07	0,200	S06-S02	0,240
S06-S04	0,455	S09-S07	0,220	S05-S07	0,200	S02-S08	0,262
S04-S03	0,557	S04-S03	0,240	S02-S09	0,240	S06-S07	0,364
S08-S07	0,583	S09-S05	0,286	S03-S08	0,352	S05-S06	0,488
S02-S03	0,594	S08-S04	0,403	S01-S08	0,352	S06-S03	0,522
S08-S01	0,616	S09-S08	0,459	S07-S02	0,393	S01-S02	0,522
S01-S03	0,709	S05-S04	0,488	S06-S02	0,455	S06-S04	0,557
S06-S08	0,720	S08-S06	0,519	S02-S08	0,519	S04-S02	0,557
S08-S02	0,720	S07-S04	0,594	S07-S03	0,594	S06-S01	0,594
S07-S03	0,749	S05-S06	0,631	S07-S01	0,594	S03-S02	0,594
S08-S04	0,756	S03-S02	0,669	S06-S01	0,669	S01-S07	0,709
S04-S07	0,790	S08-S07	0,720	S06-S03	0,669	S04-S07	0,749
S04-S01	0,831	S07-S06	0,749	S08-S09	0,685	S03-S07	0,790
S02-S07	0,831	S01-S02	0,749	S03-S02	0,749	S07-S02	0,790
S09-S05	0,873	S08-S05	0,830	S01-S02	0,749	S01-S03	0,915
S02-S01	0,873	S06-S04	0,831	S07-S06	0,915	S01-S04	0,957
S04-S02	0,957	S05-S07	0,873	S04-S05	1,000	S04-S03	0,957
S01-S07	0,957	S03-S01	0,915	S01-S03	1,000	S09-S08	0,981

DkEIL3		*DkPSY*		*DkXTH9*		*DkETR2*	
Comp.	Sig.	Comp.	Sig.	Comp.	Sig.	Comp.	Sig.
S05-S09	0,001	S02-S09	0,001	S02-S09	0,001	S05-S02	0,003
S05-S08	0,001	S01-S09	0,002	S02-S08	0,001	S05-S03	0,012
S05-S04	0,019	S03-S09	0,004	S01-S09	0,007	S04-S02	0,012
S07-S08	0,023	S02-S08	0,008	S01-S08	0,007	S05-S01	0,019
S07-S09	0,029	S01-S08	0,013	S03-S09	0,011	S05-S09	0,019
S05-S06	0,037	S02-S07	0,016	S03-S08	0,011	S07-S02	0,033
S03-S08	0,059	S03-S08	0,019	S02-S06	0,017	S04-S03	0,037
S01-S08	0,073	S01-S07	0,029	S02-S07	0,027	S06-S02	0,037
S03-S09	0,078	S03-S07	0,043	S01-S06	0,067	S08-S02	0,050
S05-S02	0,088	S04-S09	0,043	S02-S05	0,086	S04-S01	0,055
S02-S08	0,090	S02-S06	0,070	S01-S07	0,096	S04-S09	0,055
S01-S09	0,098	S05-S09	0,070	S03-S06	0,096	S07-S03	0,088
S05-S01	0,109	S01-S06	0,109	S05-S08	0,102	S06-S03	0,098
S02-S09	0,122	S04-S08	0,115	S04-S08	0,118	S08-S03	0,115
S05-S03	0,135	S02-S05	0,150	S05-S09	0,120	S07-S01	0,122
S06-S08	0,174	S03-S06	0,150	S03-S07	0,134	S07-S09	0,122
S07-S04	0,200	S06-S09	0,150	S02-S04	0,143	S06-S09	0,135
S06-S09	0,240	S05-S08	0,166	S04-S09	0,143	S06-S01	0,135
S04-S08	0,262	S02-S04	0,220	S07-S08	0,234	S08-S09	0,152
S05-S07	0,286	S01-S05	0,220	S01-S05	0,244	S08-S01	0,152
S07-S06	0,311	S04-S07	0,240	S07-S09	0,292	S05-S06	0,393
S04-S09	0,364	S03-S05	0,286	S06-S08	0,297	S05-S07	0,423
S03-S04	0,393	S06-S08	0,294	S03-S05	0,318	S05-S08	0,504
S01-S04	0,455	S01-S04	0,311	S01-S04	0,333	S09-S02	0,557
S02-S04	0,522	S05-S07	0,337	S06-S09	0,375	S01-S02	0,557
S07-S02	0,522	S03-S04	0,393	S03-S04	0,413	S05-S04	0,669
S03-S06	0,557	S07-S09	0,393	S02-S03	0,471	S04-S06	0,669
S07-S01	0,594	S04-S06	0,557	S04-S06	0,503	S03-S02	0,669
S01-S06	0,631	S06-S07	0,557	S05-S06	0,506	S04-S07	0,709
S07-S03	0,669	S07-S08	0,599	S02-S01	0,579	S04-S08	0,775
S02-S06	0,709	S02-S03	0,709	S04-S07	0,602	S01-S03	0,873
S09-S08	0,756	S05-S06	0,709	S05-S07	0,618	S09-S03	0,873
S06-S04	0,790	S08-S09	0,811	S09-S08	0,804	S08-S06	0,924
S03-S02	0,831	S02-S01	0,831	S01-S03	0,868	S07-S06	0,957
S03-S01	0,915	S04-S05	0,831	S07-S06	0,868	S08-S07	0,962
S01-S02	0,915	S01-S03	0,873	S04-S05	0,941	S01-S09	1,000

JIRO

Test: Kruskal-Wallis (p<0.05)

TRAIT / GENE	Sig.
WEIGHT	0,000
CI	0,000
FIRNESS	0,000
DkERF16	0,008
DkERF8	0,003
DkERF18	0,001
DkACO1	0,005
DkACO2	0,007
DkACS2	0,013
DkERS1	0,018
DkETR3	0,003
DkEIL1	0,062
DkEIL3	0,091
DkPSY	0,002
DkXTH9	0,013
DkETR2	0,009

Pairwise Comparisons (p<0.05)

WEIGHT Comp.	Sig.	CI Comp.	Sig.	FIRMNESS Comp.	Sig.
S3-S7	0,000	S1-S12	0,000	S13-S1	0,000
S1-S7	0,000	S2-S12	0,000	S12-S1	0,000
S2-S7	0,001	S1-S13	0,000	S13-S3	0,000
S4-S7	0,001	S3-S12	0,000	S11-S1	0,000
S3-S11	0,002	S2-S13	0,000	S13-S2	0,000
S3-S10	0,002	S1-S11	0,000	S12-S3	0,000
S1-S11	0,002	S2-S11	0,000	S12-S2	0,000
S1-S10	0,002	S3-S13	0,000	S11-S3	0,000
S3-S8	0,003	S3-S11	0,000	S11-S2	0,000
S1-S8	0,004	S4-S12	0,000	S8-S1	0,001
S2-S11	0,005	S1-S10	0,000	S13-S4	0,001
S2-S10	0,005	S2-S10	0,000	S13-S5	0,001
S3-S9	0,005	S4-S13	0,000	S10-S1	0,001
S3-S6	0,006	S5-S12	0,000	S8-S3	0,004
S4-S11	0,006	S1-S9	0,000	S12-S4	0,004
S1-S9	0,006	S3-S10	0,001	S12-S5	0,004
S4-S10	0,006	S2-S9	0,001	S6-S1	0,005
S1-S6	0,007	S4-S11	0,001	S10-S3	0,008
S2-S8	0,008	S5-S13	0,002	S9-S1	0,008
S4-S8	0,010	S1-S8	0,002	S8-S2	0,008
S2-S9	0,012	S3-S9	0,003	S11-S4	0,008
S2-S6	0,013	S6-S12	0,003	S13-S7	0,009
S4-S9	0,015	S2-S8	0,004	S11-S5	0,009
S3-S5	0,015	S5-S11	0,005	S7-S1	0,012
S4-S6	0,016	S4-S10	0,008	S13-S9	0,014
S1-S5	0,018	S6-S13	0,008	S10-S2	0,016
S12-S7	0,027	S3-S8	0,009	S13-S6	0,020
S3-S13	0,032	S7-S12	0,009	S6-S3	0,022
S2-S5	0,033	S1-S7	0,011	S12-S7	0,023
S4-S5	0,038	S2-S7	0,019	S9-S3	0,030
S1-S13	0,039	S6-S11	0,020	S12-S9	0,034
S2-S13	0,067	S7-S13	0,025	S6-S2	0,043
S4-S13	0,074	S4-S9	0,025	S7-S3	0,043
S12-S11	0,078	S5-S10	0,026	S11-S7	0,044
S12-S10	0,081	S1-S6	0,033	S12-S6	0,045
S12-S8	0,110	S3-S7	0,036	S13-S10	0,050
S12-S9	0,146	S8-S12	0,036	S5-S1	0,056
S12-S6	0,153	S7-S11	0,053	S9-S2	0,058
S13-S7	0,159	S2-S6	0,053	S4-S1	0,061
S3-S12	0,182	S4-S8	0,068	S11-S9	0,062
S1-S12	0,219	S5-S9	0,070	S7-S2	0,081
S5-S7	0,261	S6-S10	0,084	S11-S6	0,082
S12-S5	0,274	S8-S13	0,084	S13-S8	0,084
S2-S12	0,319	S3-S6	0,089	S12-S10	0,104
S4-S12	0,326	S9-S12	0,091	S8-S4	0,133
S13-S11	0,339	S1-S5	0,106	S8-S5	0,143
S13-S10	0,348	S8-S11	0,153	S5-S3	0,150
S12-S13	0,419	S2-S5	0,157	S4-S3	0,160
S13-S8	0,430	S5-S8	0,160	S12-S8	0,164
S6-S7	0,430	S7-S10	0,184	S11-S10	0,172
S9-S7	0,445	S4-S7	0,188	S10-S4	0,205
S5-S11	0,502	S6-S9	0,188	S10-S5	0,219
S5-S10	0,514	S9-S13	0,188	S5-S2	0,256
S13-S9	0,518	S10-S12	0,201	S11-S8	0,258
S13-S6	0,534	S3-S5	0,228	S4-S2	0,272
S8-S7	0,534	S1-S4	0,239	S6-S4	0,371
S5-S8	0,615	S9-S11	0,308	S8-S7	0,378
S10-S7	0,637	S2-S4	0,328	S6-S5	0,391
S11-S7	0,650	S4-S6	0,358	S2-S1	0,421
S3-S2	0,697	S7-S9	0,358	S9-S4	0,441
S5-S9	0,719	S6-S8	0,364	S8-S9	0,464
S3-S4	0,723	S10-S13	0,364	S9-S5	0,464
S5-S6	0,737	S5-S7	0,371	S10-S7	0,518
S6-S11	0,737	S8-S10	0,412	S7-S4	0,535
S6-S10	0,751	S3-S4	0,434	S8-S6	0,543
S9-S11	0,756	S11-S12	0,502	S13-S11	0,551
S9-S10	0,770	S10-S11	0,543	S7-S5	0,559
S13-S5	0,775	S7-S8	0,611	S10-S9	0,619
S1-S2	0,809	S5-S6	0,619	S3-S1	0,679
S1-S4	0,834	S4-S5	0,673	S10-S6	0,709
S6-S8	0,867	S8-S9	0,682	S2-S3	0,719
S8-S11	0,867	S9-S10	0,682	S13-S12	0,737
S3-S1	0,875	S6-S7	0,691	S6-S7	0,785
S8-S10	0,881	S13-S12	0,709	S12-S11	0,794
S9-S8	0,886	S1-S3	0,715	S8-S10	0,813
S6-S9	0,980	S11-S13	0,766	S9-S7	0,881
S4-S2	0,982	S1-S2	0,836	S6-S9	0,901
S10-S11	0,985	S2-S3	0,868	S5-S4	0,970

DkERF16 Comp.	Sig.	*DkERF8* Comp.	Sig.	*DkERF18* Comp.	Sig.	*DkACO1* Comp.	Sig.
S10-S12	0,001	S02-S13	0,000	S08-S02	0,001	S13-S01	0,003
S10-S13	0,001	S02-S12	0,001	S08-S03	0,001	S13-S02	0,005
S06-S12	0,001	S04-S13	0,002	S08-S01	0,001	S11-S01	0,005
S06-S13	0,002	S04-S12	0,003	S10-S02	0,004	S08-S01	0,005
S09-S12	0,008	S01-S13	0,004	S10-S01	0,005	S13-S04	0,005
S05-S12	0,009	S05-S13	0,006	S08-S12	0,005	S08-S02	0,007
S10-S02	0,012	S01-S12	0,006	S10-S03	0,005	S11-S02	0,007
S09-S13	0,012	S02-S11	0,007	S08-S13	0,006	S11-S04	0,008
S05-S13	0,013	S05-S12	0,010	S09-S02	0,007	S08-S04	0,008
S07-S12	0,015	S03-S13	0,015	S09-S01	0,007	S10-S01	0,011
S06-S02	0,016	S04-S11	0,023	S09-S03	0,008	S13-S03	0,017
S07-S13	0,020	S03-S12	0,025	S04-S02	0,010	S10-S02	0,017
S09-S02	0,069	S02-S06	0,028	S04-S01	0,010	S10-S04	0,019
S08-S12	0,069	S02-S07	0,030	S04-S03	0,012	S05-S01	0,023
S10-S04	0,075	S01-S11	0,035	S10-S12	0,019	S08-S03	0,026
S05-S02	0,075	S10-S13	0,047	S10-S13	0,021	S11-S03	0,026
S10-S03	0,083	S02-S08	0,056	S09-S12	0,028	S07-S01	0,032
S08-S13	0,088	S05-S11	0,061	S09-S13	0,030	S05-S02	0,038
S06-S04	0,096	S09-S13	0,066	S07-S01	0,038	S05-S04	0,042
S06-S03	0,103	S10-S12	0,072	S04-S12	0,040	S06-S01	0,045
S07-S02	0,104	S02-S09	0,078	S04-S13	0,043	S10-S03	0,054
S10-S01	0,115	S04-S06	0,078	S07-S02	0,043	S07-S02	0,054
S04-S12	0,121	S04-S07	0,084	S07-S03	0,052	S07-S04	0,059
S10-S08	0,131	S08-S13	0,091	S05-S01	0,061	S13-S12	0,076
S11-S12	0,132	S09-S12	0,098	S05-S02	0,072	S06-S02	0,076
S10-S11	0,141	S01-S06	0,100	S08-S11	0,078	S06-S04	0,083
S06-S01	0,141	S02-S10	0,106	S08-S06	0,078	S09-S01	0,096
S04-S13	0,152	S01-S07	0,107	S05-S03	0,084	S08-S12	0,105
S11-S13	0,160	S03-S11	0,123	S06-S01	0,100	S11-S12	0,105
S01-S12	0,160	S08-S12	0,132	S11-S01	0,100	S05-S03	0,105
S06-S08	0,163	S04-S08	0,142	S11-S02	0,123	S07-S03	0,141
S06-S11	0,171	S07-S13	0,152	S06-S02	0,123	S09-S02	0,162
S01-S13	0,194	S06-S13	0,163	S08-S05	0,132	S09-S04	0,172
S03-S12	0,212	S01-S08	0,168	S07-S12	0,132	S12-S01	0,172
S03-S13	0,253	S05-S06	0,174	S07-S13	0,142	S10-S12	0,187
S09-S03	0,267	S04-S09	0,186	S06-S03	0,142	S06-S03	0,187
S09-S04	0,278	S05-S07	0,186	S11-S03	0,142	S13-S09	0,258
S05-S03	0,283	S07-S12	0,212	S10-S06	0,186	S13-S06	0,291
S05-S04	0,295	S01-S09	0,212	S10-S11	0,186	S12-S02	0,291
S08-S02	0,314	S06-S12	0,225	S08-S07	0,199	S05-S12	0,309
S09-S01	0,341	S02-S03	0,240	S05-S12	0,199	S12-S04	0,309
S07-S03	0,349	S04-S10	0,240	S05-S13	0,212	S11-S09	0,320
S05-S01	0,358	S01-S10	0,264	S09-S11	0,240	S08-S09	0,320
S10-S07	0,373	S10-S11	0,270	S09-S06	0,240	S09-S03	0,320
S07-S04	0,373	S05-S08	0,287	S10-S05	0,287	S13-S07	0,365
S09-S11	0,396	S03-S06	0,304	S04-S06	0,304	S11-S06	0,365
S05-S11	0,415	S03-S07	0,321	S04-S11	0,304	S08-S06	0,365
S09-S08	0,416	S09-S11	0,340	S06-S12	0,304	S07-S12	0,386
S02-S12	0,416	S05-S09	0,358	S11-S12	0,304	S03-S01	0,409
S07-S01	0,436	S11-S13	0,378	S06-S13	0,321	S13-S05	0,451
S11-S02	0,436	S02-S05	0,398	S11-S13	0,321	S11-S07	0,451
S06-S07	0,438	S08-S11	0,419	S09-S05	0,358	S08-S07	0,451
S05-S08	0,438	S05-S10	0,440	S10-S07	0,398	S10-S09	0,468
S10-S05	0,462	S04-S03	0,463	S04-S05	0,440	S06-S12	0,474
S04-S02	0,462	S03-S08	0,463	S13-S01	0,450	S11-S05	0,546
S10-S09	0,485	S01-S03	0,470	S08-S04	0,463	S08-S05	0,546
S02-S13	0,485	S11-S12	0,485	S12-S01	0,470	S10-S06	0,546
S07-S11	0,499	S03-S09	0,557	S09-S07	0,485	S12-S03	0,546
S01-S02	0,499	S10-S06	0,557	S08-S09	0,557	S05-S09	0,649
S06-S05	0,535	S10-S07	0,582	S04-S07	0,582	S09-S12	0,649
S07-S08	0,535	S07-S11	0,582	S13-S02	0,582	S04-S01	0,649
S06-S09	0,561	S06-S11	0,607	S12-S02	0,607	S03-S02	0,651
S03-S02	0,603	S02-S04	0,659	S07-S11	0,633	S13-S10	0,651
S08-S03	0,703	S03-S10	0,659	S07-S06	0,633	S10-S07	0,651
S08-S04	0,786	S09-S06	0,659	S13-S03	0,633	S02-S01	0,673
S11-S03	0,812	S01-S05	0,669	S08-S10	0,659	S03-S04	0,678
S08-S01	0,822	S04-S05	0,686	S12-S03	0,659	S07-S09	0,749
S09-S07	0,846	S09-S07	0,686	S03-S01	0,742	S11-S10	0,763
S05-S07	0,877	S05-S03	0,741	S10-S04	0,769	S08-S10	0,763
S01-S03	0,887	S02-S01	0,742	S02-S01	0,793	S10-S05	0,763
S04-S03	0,890	S10-S08	0,769	S05-S06	0,797	S05-S06	0,763
S08-S11	0,903	S08-S06	0,769	S05-S11	0,797	S06-S09	0,853
S11-S04	0,903	S08-S07	0,797	S07-S05	0,826	S05-S07	0,880
S10-S06	0,907	S12-S13	0,854	S10-S09	0,883	S07-S06	0,880
S13-S12	0,907	S10-S09	0,883	S09-S04	0,883	S13-S11	0,880
S11-S01	0,924	S09-S08	0,883	S03-S02	0,941	S13-S08	0,880
S09-S05	0,969	S01-S04	0,948	S13-S12	0,971	S04-S02	0,970
S01-S04	0,986	S07-S06	0,971	S06-S11	1,000	S08-S11	1,000

DkACO2 Comp.	Sig.	*DkACS2* Comp.	Sig.	*DkERS1* Comp.	Sig.	*DkETR3* Comp.	Sig.
S10-S02	0,001	S13-S04	0,002	S10-S02	0,003	S04-S13	0,000
S10-S01	0,002	S08-S04	0,004	S10-S01	0,003	S04-S12	0,000
S05-S02	0,003	S13-S02	0,006	S13-S01	0,007	S05-S13	0,005
S10-S03	0,003	S10-S04	0,007	S10-S03	0,007	S10-S13	0,005
S05-S01	0,006	S13-S07	0,010	S13-S02	0,007	S05-S12	0,007
S06-S02	0,008	S08-S02	0,011	S11-S01	0,013	S04-S11	0,007
S05-S03	0,010	S12-S04	0,012	S08-S01	0,013	S10-S12	0,007
S13-S02	0,011	S08-S07	0,019	S08-S02	0,014	S07-S13	0,007
S06-S01	0,014	S10-S02	0,019	S11-S02	0,014	S04-S01	0,009
S11-S02	0,017	S06-S04	0,028	S13-S03	0,015	S07-S12	0,010
S13-S01	0,019	S13-S01	0,028	S08-S03	0,028	S08-S13	0,017
S06-S03	0,025	S10-S07	0,030	S11-S03	0,028	S06-S13	0,019
S11-S01	0,027	S12-S02	0,033	S05-S01	0,042	S08-S12	0,023
S10-S12	0,030	S08-S01	0,045	S06-S01	0,042	S06-S12	0,025
S13-S03	0,033	S13-S09	0,052	S09-S01	0,042	S04-S03	0,030
S08-S02	0,036	S13-S05	0,052	S09-S02	0,052	S04-S02	0,030
S10-S07	0,043	S12-S07	0,052	S06-S02	0,052	S05-S11	0,056
S11-S03	0,047	S10-S01	0,066	S05-S02	0,052	S09-S13	0,056
S09-S02	0,047	S06-S02	0,066	S10-S04	0,056	S05-S01	0,059
S08-S01	0,051	S13-S11	0,066	S10-S07	0,072	S10-S11	0,061
S09-S01	0,063	S03-S04	0,072	S12-S01	0,076	S10-S01	0,063
S05-S12	0,072	S08-S05	0,084	S05-S03	0,091	S09-S12	0,072
S04-S02	0,078	S08-S09	0,084	S09-S03	0,091	S07-S11	0,078
S08-S03	0,091	S06-S07	0,098	S06-S03	0,091	S07-S01	0,079
S04-S01	0,097	S12-S01	0,100	S13-S04	0,098	S04-S09	0,084
S05-S07	0,098	S08-S11	0,106	S12-S02	0,098	S08-S01	0,135
S10-S04	0,114	S10-S05	0,123	S13-S07	0,123	S08-S11	0,142
S09-S03	0,114	S10-S09	0,123	S08-S04	0,152	S02-S13	0,142
S06-S12	0,142	S10-S11	0,152	S11-S04	0,152	S03-S13	0,142
S10-S09	0,174	S03-S02	0,152	S12-S03	0,163	S06-S01	0,144
S13-S12	0,174	S06-S01	0,168	S08-S07	0,186	S06-S11	0,152
S04-S03	0,174	S13-S03	0,186	S11-S07	0,186	S05-S03	0,174
S06-S07	0,186	S12-S05	0,186	S07-S01	0,189	S05-S02	0,174
S07-S02	0,186	S12-S09	0,186	S10-S12	0,199	S02-S12	0,174
S07-S01	0,206	S11-S04	0,199	S04-S01	0,224	S03-S12	0,174
S10-S08	0,212	S03-S07	0,212	S07-S02	0,255	S10-S02	0,186
S05-S04	0,225	S12-S11	0,225	S13-S12	0,304	S10-S03	0,186
S13-S07	0,225	S09-S04	0,240	S04-S02	0,304	S04-S06	0,199
S11-S12	0,225	S05-S04	0,240	S10-S09	0,321	S04-S08	0,212
S12-S02	0,240	S08-S03	0,270	S10-S06	0,321	S07-S02	0,225
S12-S01	0,257	S06-S05	0,304	S10-S05	0,321	S07-S03	0,225
S11-S07	0,287	S06-S09	0,304	S06-S04	0,358	S09-S01	0,286
S05-S09	0,321	S03-S01	0,308	S05-S04	0,358	S09-S11	0,321
S10-S11	0,340	S13-S06	0,358	S09-S04	0,358	S04-S07	0,340
S08-S12	0,358	S10-S03	0,358	S07-S03	0,378	S05-S09	0,358
S07-S03	0,358	S06-S11	0,358	S08-S12	0,419	S08-S02	0,358
S05-S08	0,378	S11-S02	0,358	S11-S12	0,419	S08-S03	0,358
S06-S04	0,378	S09-S02	0,419	S05-S07	0,419	S11-S13	0,358
S10-S13	0,419	S05-S02	0,419	S06-S07	0,419	S10-S09	0,378
S09-S12	0,419	S11-S07	0,463	S09-S07	0,419	S06-S02	0,378
S13-S04	0,440	S08-S06	0,485	S04-S03	0,440	S06-S03	0,378
S08-S07	0,440	S12-S03	0,485	S13-S09	0,463	S04-S10	0,398
S12-S03	0,440	S13-S12	0,532	S13-S06	0,463	S04-S05	0,419
S10-S06	0,485	S03-S05	0,532	S13-S05	0,463	S11-S12	0,419
S06-S09	0,508	S03-S09	0,532	S12-S04	0,532	S07-S09	0,440
S09-S07	0,508	S09-S07	0,532	S03-S01	0,599	S03-S01	0,501
S11-S04	0,532	S05-S07	0,532	S08-S06	0,607	S02-S01	0,501
S05-S11	0,557	S01-S04	0,554	S11-S06	0,607	S01-S13	0,522
S04-S12	0,557	S11-S01	0,576	S08-S09	0,607	S02-S11	0,582
S06-S08	0,582	S07-S04	0,582	S11-S09	0,607	S03-S11	0,582
S13-S09	0,582	S10-S06	0,607	S08-S05	0,607	S01-S12	0,588
S03-S01	0,657	S03-S11	0,607	S11-S05	0,607	S05-S06	0,633
S13-S08	0,659	S09-S01	0,646	S12-S07	0,607	S08-S09	0,633
S05-S13	0,659	S05-S01	0,646	S10-S08	0,633	S06-S09	0,659
S04-S07	0,659	S13-S10	0,686	S10-S11	0,633	S05-S08	0,659
S11-S09	0,686	S08-S12	0,686	S02-S01	0,767	S10-S06	0,659
S03-S02	0,686	S06-S03	0,686	S05-S12	0,769	S09-S03	0,659
S10-S05	0,713	S02-S04	0,713	S09-S12	0,769	S09-S02	0,659
S05-S06	0,741	S12-S06	0,769	S06-S12	0,769	S10-S08	0,686
S08-S04	0,741	S01-S02	0,793	S10-S13	0,797	S07-S06	0,741
S11-S08	0,769	S13-S08	0,826	S03-S02	0,797	S07-S08	0,769
S06-S11	0,797	S10-S12	0,826	S13-S08	0,826	S11-S01	0,857
S09-S04	0,826	S08-S10	0,854	S13-S11	0,826	S05-S07	0,883
S13-S11	0,883	S07-S02	0,854	S07-S04	0,912	S10-S07	0,912
S07-S12	0,883	S11-S05	0,912	S08-S11	1,000	S12-S13	0,912
S06-S13	0,912	S11-S09	0,912	S05-S09	1,000	S05-S10	0,971
S08-S09	0,912	S01-S07	0,921	S05-S06	1,000	S08-S06	0,971
S02-S01	0,935	S05-S09	1,000	S06-S09	1,000	S02-S03	1,000

DkEIL1 Comp.	Sig.	*DkEIL3* Comp.	Sig.	*DkPSY* Comp.	Sig.	*DkXTH9* Comp.	Sig.	*DkETR2* Comp.	Sig.
S10-S13	0,004	S10-S12	0,009	S01-S13	0,001	S03-S12	0,001	S04-S13	0,000
S10-S12	0,005	S10-S01	0,010	S02-S13	0,001	S03-S13	0,001	S04-S12	0,001
S04-S13	0,010	S05-S12	0,012	S04-S13	0,002	S02-S12	0,002	S05-S13	0,007
S04-S12	0,014	S05-S01	0,014	S01-S12	0,002	S10-S12	0,003	S05-S12	0,007
S08-S13	0,019	S10-S13	0,017	S02-S12	0,003	S05-S12	0,004	S06-S13	0,008
S10-S11	0,023	S05-S13	0,023	S04-S12	0,003	S02-S13	0,005	S10-S13	0,008
S08-S12	0,025	S08-S01	0,035	S01-S11	0,008	S10-S13	0,007	S06-S12	0,009
S05-S13	0,025	S08-S12	0,036	S03-S13	0,009	S05-S13	0,009	S10-S12	0,009
S05-S12	0,033	S10-S03	0,047	S06-S13	0,012	S08-S12	0,036	S08-S13	0,011
S10-S09	0,036	S10-S02	0,052	S02-S11	0,014	S11-S12	0,043	S08-S12	0,012
S02-S13	0,043	S05-S03	0,061	S01-S09	0,016	S03-S04	0,047	S04-S01	0,016
S04-S11	0,052	S08-S13	0,061	S04-S11	0,017	S03-S07	0,047	S04-S03	0,019
S02-S12	0,056	S06-S01	0,061	S03-S12	0,017	S03-S06	0,056	S04-S02	0,047
S04-S09	0,078	S05-S02	0,066	S10-S13	0,017	S03-S09	0,061	S04-S11	0,056
S08-S11	0,084	S06-S12	0,066	S06-S12	0,023	S08-S13	0,066	S07-S13	0,056
S10-S03	0,106	S06-S13	0,106	S01-S08	0,028	S11-S13	0,078	S07-S12	0,061
S05-S11	0,106	S09-S01	0,107	S02-S09	0,030	S01-S12	0,100	S09-S13	0,066
S08-S09	0,123	S09-S12	0,123	S10-S12	0,030	S02-S04	0,114	S09-S12	0,072
S06-S13	0,142	S10-S04	0,132	S04-S09	0,036	S02-S07	0,114	S05-S01	0,085
S07-S13	0,142	S08-S03	0,142	S01-S07	0,042	S09-S12	0,114	S06-S01	0,097
S10-S06	0,152	S08-S02	0,152	S02-S08	0,052	S06-S12	0,123	S10-S01	0,097
S10-S07	0,152	S07-S01	0,158	S04-S08	0,061	S02-S06	0,132	S04-S09	0,098
S05-S09	0,152	S05-S04	0,163	S05-S13	0,066	S02-S09	0,142	S04-S07	0,114
S02-S11	0,163	S10-S11	0,174	S03-S11	0,066	S10-S07	0,142	S05-S03	0,114
S06-S12	0,174	S11-S01	0,178	S02-S07	0,078	S10-S04	0,142	S11-S13	0,114
S07-S12	0,174	S09-S13	0,186	S06-S11	0,084	S04-S12	0,142	S08-S01	0,119
S01-S13	0,183	S07-S12	0,186	S01-S05	0,088	S07-S12	0,142	S11-S12	0,123
S04-S03	0,199	S10-S07	0,199	S04-S07	0,091	S03-S01	0,148	S06-S03	0,132
S03-S13	0,199	S05-S11	0,212	S10-S11	0,106	S03-S11	0,152	S10-S03	0,132
S10-S01	0,206	S11-S12	0,212	S05-S12	0,106	S01-S13	0,158	S02-S13	0,132
S01-S12	0,218	S04-S01	0,224	S03-S09	0,123	S10-S06	0,163	S02-S12	0,142
S02-S09	0,225	S06-S03	0,225	S07-S13	0,142	S05-S04	0,163	S08-S03	0,163
S03-S12	0,240	S05-S07	0,240	S06-S09	0,152	S05-S07	0,163	S05-S02	0,225
S04-S07	0,270	S06-S02	0,240	S02-S05	0,163	S03-S08	0,174	S05-S11	0,255
S04-S06	0,270	S07-S13	0,270	S04-S05	0,186	S10-S09	0,174	S06-S02	0,255
S08-S03	0,287	S04-S12	0,270	S03-S08	0,186	S05-S06	0,186	S10-S02	0,255
S04-S01	0,332	S10-S09	0,287	S10-S09	0,186	S09-S13	0,186	S03-S13	0,255
S05-S03	0,340	S11-S13	0,304	S08-S13	0,199	S05-S09	0,199	S03-S12	0,270
S10-S02	0,378	S08-S04	0,321	S07-S12	0,212	S06-S13	0,199	S06-S11	0,287
S08-S06	0,378	S05-S09	0,340	S01-S10	0,224	S04-S13	0,225	S10-S11	0,287
S08-S07	0,378	S09-S03	0,358	S06-S08	0,225	S07-S13	0,225	S08-S02	0,304
S07-S11	0,398	S09-S02	0,378	S03-S07	0,255	S02-S01	0,278	S07-S01	0,316
S06-S11	0,398	S04-S13	0,378	S01-S06	0,264	S02-S11	0,304	S04-S08	0,340
S09-S13	0,419	S08-S11	0,398	S10-S08	0,270	S10-S01	0,324	S08-S11	0,340
S08-S01	0,440	S02-S01	0,411	S05-S11	0,287	S02-S08	0,340	S09-S01	0,349
S01-S11	0,440	S03-S01	0,430	S08-S12	0,287	S05-S01	0,358	S05-S09	0,378
S05-S06	0,440	S10-S06	0,440	S09-S13	0,287	S10-S11	0,358	S04-S10	0,398
S05-S07	0,440	S08-S07	0,440	S06-S07	0,304	S10-S08	0,398	S04-S06	0,398
S02-S03	0,463	S06-S04	0,463	S01-S03	0,308	S05-S11	0,398	S05-S07	0,419
S09-S12	0,485	S07-S03	0,485	S10-S07	0,358	S05-S08	0,440	S06-S09	0,419
S05-S01	0,501	S05-S06	0,508	S02-S10	0,398	S08-S07	0,532	S10-S09	0,419
S10-S05	0,508	S07-S02	0,508	S09-S12	0,398	S08-S04	0,532	S04-S05	0,440
S07-S09	0,508	S02-S12	0,508	S04-S10	0,440	S03-S05	0,557	S07-S03	0,440
S06-S09	0,508	S11-S03	0,532	S03-S05	0,440	S08-S06	0,582	S10-S07	0,463
S03-S11	0,508	S03-S12	0,532	S05-S09	0,440	S11-S07	0,582	S06-S07	0,463
S11-S13	0,532	S06-S11	0,557	S11-S13	0,440	S11-S04	0,582	S11-S01	0,480
S01-S09	0,543	S11-S02	0,557	S02-S06	0,463	S03-S10	0,607	S01-S13	0,480
S10-S08	0,582	S08-S09	0,582	S07-S11	0,485	S08-S09	0,607	S08-S09	0,485
S04-S02	0,582	S10-S08	0,607	S04-S06	0,508	S11-S06	0,633	S09-S03	0,485
S02-S06	0,582	S06-S07	0,607	S06-S05	0,508	S11-S09	0,659	S01-S12	0,501
S02-S07	0,582	S04-S03	0,633	S02-S03	0,532	S03-S02	0,686	S02-S01	0,522
S11-S12	0,607	S09-S04	0,659	S04-S03	0,582	S01-S07	0,742	S08-S07	0,532
S03-S09	0,633	S04-S02	0,659	S10-S05	0,582	S01-S04	0,742	S11-S03	0,659
S02-S01	0,634	S02-S13	0,659	S05-S08	0,582	S01-S06	0,793	S07-S02	0,686
S04-S05	0,741	S13-S01	0,669	S11-S12	0,582	S13-S12	0,797	S02-S03	0,713
S08-S02	0,741	S05-S08	0,686	S01-S04	0,599	S08-S01	0,818	S07-S11	0,741
S10-S04	0,741	S03-S13	0,686	S08-S11	0,607	S01-S09	0,818	S09-S02	0,741
S04-S08	0,826	S06-S09	0,769	S01-S02	0,646	S02-S05	0,854	S03-S01	0,755
S05-S02	0,826	S09-S11	0,769	S07-S09	0,686	S11-S01	0,870	S09-S11	0,797
S07-S03	0,854	S08-S06	0,797	S05-S07	0,713	S02-S10	0,912	S05-S08	0,854
S06-S03	0,854	S12-S01	0,818	S09-S11	0,769	S09-S04	0,912	S10-S08	0,912
S09-S11	0,854	S09-S07	0,826	S03-S10	0,826	S09-S07	0,912	S06-S08	0,912
S01-S03	0,857	S07-S04	0,826	S08-S09	0,826	S10-S05	0,941	S05-S06	0,941
S08-S05	0,912	S13-S12	0,826	S12-S13	0,826	S08-S11	0,941	S05-S10	0,941
S12-S13	0,912	S11-S04	0,883	S07-S08	0,854	S06-S04	0,941	S07-S09	0,941
S01-S06	0,987	S10-S05	0,912	S03-S06	0,912	S06-S07	0,941	S11-S02	0,941
S01-S07	0,987	S07-S11	0,941	S06-S10	0,912	S09-S06	0,971	S12-S13	0,971
S06-S07	1,000	S02-S03	0,971	S02-S04	0,941	S04-S07	1,000	S06-S10	1,000

TAKURA

Test: Kruskal-Wallis (p<0.05)

TRAIT / GENE	Sig.
WEIGHT	0,000
CI	0,000
FIRNESS	0,000
DkERF16	0,085
DkERF8	0,078
DkERF18	0,006
DkACO1	0,089
DkACO2	0,005
DkACS2	0,011
DkERS1	0,013
DkETR3	0,414
DkEIL1	0,303
DkEIL3	0,172
DkPSY	0,036
DkXTH9	0,231
DkETR2	0,019

Pairwise Comparisons (p<0.05)

WEIGHT Comp.	Sig.	CI Comp.	Sig.	FIRMNESS Comp.	Sig.
S2-S6	0,000	S1-S12	0,000	S13-S1	0,000
S2-S10	0,000	S1-S13	0,000	S12-S1	0,000
S1-S6	0,000	S1-S11	0,000	S13-S2	0,000
S2-S9	0,000	S2-S12	0,000	S12-S2	0,000
S1-S10	0,000	S2-S13	0,000	S11-S1	0,000
S2-S13	0,000	S2-S11	0,000	S11-S2	0,000
S2-S7	0,000	S1-S10	0,000	S13-S3	0,000
S2-S8	0,000	S2-S10	0,000	S12-S3	0,000
S1-S9	0,000	S3-S12	0,000	S10-S1	0,000
S1-S13	0,000	S3-S13	0,000	S13-S4	0,000
S1-S7	0,000	S3-S11	0,000	S10-S2	0,000
S1-S8	0,000	S1-S9	0,000	S11-S3	0,000
S3-S6	0,001	S2-S9	0,000	S12-S4	0,000
S3-S10	0,001	S4-S12	0,000	S9-S1	0,000
S3-S9	0,002	S4-S13	0,000	S11-S4	0,001
S2-S12	0,003	S3-S10	0,001	S9-S2	0,001
S2-S11	0,004	S4-S11	0,001	S10-S3	0,001
S3-S13	0,004	S1-S8	0,002	S10-S4	0,005
S3-S7	0,005	S5-S12	0,002	S9-S3	0,005
S3-S8	0,005	S5-S13	0,002	S13-S7	0,006
S1-S12	0,007	S4-S10	0,003	S13-S8	0,006
S1-S11	0,009	S5-S11	0,003	S13-S5	0,008
S4-S6	0,017	S2-S8	0,003	S12-S7	0,009
S5-S6	0,025	S3-S9	0,004	S12-S8	0,009
S4-S10	0,025	S1-S7	0,007	S6-S1	0,009
S5-S10	0,036	S6-S12	0,009	S12-S5	0,011
S2-S5	0,038	S6-S13	0,009	S9-S4	0,014
S3-S12	0,043	S5-S10	0,010	S13-S6	0,014
S4-S9	0,044	S6-S11	0,012	S5-S1	0,016
S3-S11	0,053	S2-S7	0,013	S6-S2	0,018
S2-S4	0,053	S4-S9	0,017	S12-S6	0,020
S5-S9	0,061	S3-S8	0,024	S7-S1	0,021
S4-S13	0,063	S1-S6	0,025	S8-S1	0,021
S1-S5	0,066	S7-S12	0,030	S11-S7	0,021
S4-S7	0,070	S7-S13	0,031	S11-S8	0,021
S4-S8	0,072	S6-S10	0,035	S11-S5	0,027
S5-S13	0,085	S7-S11	0,040	S5-S2	0,032
S1-S4	0,089	S2-S6	0,045	S7-S2	0,040
S5-S7	0,094	S5-S9	0,053	S8-S2	0,040
S5-S8	0,097	S3-S7	0,070	S11-S6	0,045
S11-S6	0,145	S4-S8	0,070	S6-S3	0,072
S12-S6	0,170	S1-S5	0,074	S10-S7	0,091
S11-S10	0,187	S8-S12	0,087	S10-S8	0,091
S12-S10	0,218	S8-S13	0,089	S10-S5	0,109
S3-S5	0,247	S7-S10	0,097	S5-S3	0,113
S11-S9	0,274	S8-S11	0,110	S8-S3	0,134
S3-S4	0,308	S2-S5	0,124	S7-S3	0,134
S4-S12	0,314	S6-S9	0,138	S6-S4	0,148
S12-S9	0,314	S3-S6	0,174	S13-S9	0,150
S11-S13	0,345	S4-S7	0,174	S10-S6	0,164
S4-S11	0,358	S5-S8	0,174	S12-S9	0,186
S11-S7	0,372	S1-S4	0,182	S9-S8	0,191
S11-S8	0,379	S8-S10	0,227	S9-S7	0,191
S2-S3	0,382	S9-S12	0,258	S5-S4	0,218
S5-S12	0,385	S9-S13	0,263	S9-S5	0,222
S12-S13	0,392	S2-S4	0,285	S4-S1	0,242
S12-S7	0,421	S7-S9	0,302	S8-S4	0,252
S12-S8	0,428	S9-S11	0,308	S7-S4	0,252
S5-S11	0,435	S3-S5	0,365	S13-S10	0,291
S1-S3	0,497	S4-S6	0,365	S9-S6	0,314
S8-S6	0,563	S5-S7	0,365	S11-S9	0,320
S7-S6	0,571	S6-S8	0,365	S12-S10	0,349
S13-S6	0,606	S1-S3	0,379	S4-S2	0,389
S8-S10	0,660	S9-S10	0,529	S3-S1	0,413
S7-S10	0,669	S2-S3	0,549	S11-S10	0,542
S13-S10	0,706	S8-S9	0,563	S3-S2	0,620
S9-S6	0,715	S10-S12	0,615	S13-S11	0,655
S9-S10	0,821	S10-S13	0,624	S10-S9	0,701
S8-S9	0,831	S3-S4	0,651	S2-S1	0,724
S7-S9	0,840	S4-S5	0,651	S4-S3	0,725
S2-S1	0,865	S5-S6	0,651	S12-S11	0,744
S13-S9	0,880	S6-S7	0,651	S6-S7	0,763
S4-S5	0,890	S7-S8	0,651	S6-S8	0,763
S10-S6	0,890	S10-S11	0,697	S6-S5	0,831
S11-S12	0,930	S1-S2	0,753	S13-S12	0,905
S8-S13	0,950	S11-S12	0,910	S5-S7	0,930
S7-S13	0,960	S11-S13	0,920	S5-S8	0,930
S8-S7	0,990	S13-S12	0,990	S7-S8	1,000

DkERF16 Comp.	Sig.	*DkERF8* Comp.	Sig.	*DkERF18* Comp.	Sig.	*DkACO1* Comp.	Sig.
S05-S11	0,006	S03-S11	0,002	S09-S01	0,001	S08-S04	0,004
S05-S10	0,008	S03-S09	0,010	S09-S03	0,002	S08-S06	0,008
S06-S11	0,009	S02-S11	0,013	S09-S02	0,002	S08-S11	0,012
S06-S10	0,012	S01-S11	0,021	S08-S01	0,002	S03-S04	0,015
S08-S11	0,019	S03-S10	0,026	S08-S03	0,004	S03-S06	0,026
S08-S10	0,024	S04-S11	0,026	S08-S02	0,005	S08-S05	0,037
S05-S09	0,074	S06-S11	0,045	S09-S04	0,020	S03-S11	0,037
S04-S11	0,098	S02-S09	0,050	S09-S10	0,025	S08-S01	0,056
S06-S09	0,098	S05-S11	0,061	S06-S01	0,029	S02-S04	0,090
S05-S07	0,106	S03-S08	0,074	S08-S04	0,035	S03-S05	0,098
S03-S11	0,107	S01-S09	0,074	S08-S10	0,043	S07-S04	0,115
S04-S10	0,117	S04-S09	0,090	S05-S01	0,047	S08-S10	0,117
S03-S10	0,128	S07-S11	0,106	S06-S03	0,048	S03-S01	0,139
S02-S11	0,128	S02-S10	0,107	S06-S02	0,059	S02-S06	0,139
S06-S07	0,134	S06-S09	0,139	S11-S01	0,068	S08-S09	0,151
S02-S10	0,151	S01-S10	0,151	S05-S03	0,080	S09-S04	0,151
S08-S09	0,164	S04-S10	0,177	S07-S01	0,088	S07-S06	0,167
S01-S11	0,164	S05-S09	0,177	S05-S02	0,097	S02-S11	0,177
S05-S01	0,177	S08-S11	0,192	S09-S11	0,116	S10-S04	0,192
S01-S10	0,192	S03-S05	0,223	S11-S03	0,116	S07-S11	0,206
S08-S07	0,206	S02-S08	0,240	S11-S02	0,138	S09-S06	0,223
S05-S02	0,223	S03-S07	0,251	S07-S03	0,148	S08-S02	0,240
S06-S01	0,223	S07-S09	0,251	S09-S05	0,164	S03-S10	0,258
S05-S03	0,258	S06-S10	0,258	S09-S07	0,172	S09-S11	0,277
S05-S04	0,277	S03-S06	0,277	S07-S02	0,172	S10-S06	0,277
S06-S02	0,277	S01-S08	0,317	S08-S11	0,178	S03-S09	0,317
S06-S03	0,317	S05-S10	0,317	S10-S01	0,221	S08-S07	0,321
S06-S04	0,338	S04-S08	0,361	S09-S06	0,243	S10-S11	0,338
S08-S01	0,338	S03-S04	0,384	S08-S05	0,243	S01-S04	0,338
S09-S11	0,338	S10-S11	0,384	S06-S04	0,243	S02-S05	0,361
S09-S10	0,384	S07-S10	0,403	S08-S07	0,244	S07-S05	0,381
S07-S11	0,403	S03-S01	0,433	S04-S01	0,252	S05-S04	0,433
S08-S02	0,408	S08-S09	0,433	S06-S10	0,281	S03-S02	0,459
S07-S10	0,448	S06-S08	0,486	S08-S06	0,346	S02-S01	0,459
S08-S03	0,459	S03-S02	0,542	S05-S04	0,346	S01-S06	0,459
S08-S04	0,486	S02-S05	0,542	S10-S03	0,369	S07-S01	0,471
S04-S09	0,486	S02-S07	0,546	S05-S10	0,394	S09-S05	0,514
S03-S09	0,514	S05-S08	0,572	S10-S02	0,419	S01-S11	0,542
S04-S07	0,521	S09-S11	0,602	S04-S03	0,419	S03-S07	0,546
S03-S07	0,546	S02-S06	0,632	S11-S04	0,445	S05-S06	0,572
S02-S09	0,572	S01-S07	0,654	S07-S04	0,470	S10-S05	0,602
S02-S07	0,599	S07-S08	0,654	S04-S02	0,472	S09-S01	0,632
S01-S09	0,663	S01-S05	0,663	S11-S10	0,501	S08-S03	0,663
S01-S07	0,683	S08-S10	0,663	S07-S10	0,520	S05-S11	0,663
S05-S08	0,695	S04-S07	0,712	S02-S01	0,616	S07-S10	0,683
S06-S08	0,794	S04-S05	0,728	S03-S01	0,673	S02-S10	0,695
S04-S01	0,794	S10-S09	0,728	S06-S11	0,686	S10-S01	0,728
S03-S01	0,828	S01-S06	0,761	S06-S07	0,748	S11-S04	0,728
S05-S06	0,896	S02-S04	0,794	S09-S08	0,822	S07-S09	0,770
S04-S02	0,896	S04-S06	0,828	S06-S05	0,822	S02-S09	0,794
S02-S01	0,896	S06-S07	0,861	S05-S11	0,857	S06-S04	0,828
S03-S02	0,931	S02-S01	0,862	S05-S07	0,904	S01-S05	0,862
S10-S11	0,931	S06-S05	0,896	S10-S04	0,928	S09-S10	0,896
S04-S03	0,965	S01-S04	0,931	S02-S03	0,928	S11-S06	0,896
S09-S07	0,984	S05-S07	0,953	S07-S11	0,968	S07-S02	0,953

DkACO2 Comp.	Sig.	*DkACS2* Comp.	Sig.	*DkERS1* Comp.	Sig.	*DkETR3* Comp.	Sig.
S08-S01	0,002	S08-S04	0,000	S08-S01	0,001	S06-S01	0,013
S10-S01	0,003	S08-S05	0,001	S08-S05	0,003	S08-S01	0,033
S11-S01	0,004	S10-S04	0,004	S09-S01	0,007	S04-S01	0,068
S08-S03	0,008	S08-S03	0,008	S08-S03	0,008	S06-S11	0,074
S10-S03	0,009	S10-S05	0,013	S08-S02	0,009	S06-S02	0,074
S09-S01	0,010	S09-S04	0,013	S09-S05	0,013	S10-S01	0,107
S11-S03	0,012	S08-S06	0,017	S11-S01	0,017	S06-S09	0,128
S08-S02	0,019	S08-S01	0,030	S08-S04	0,021	S06-S05	0,128
S10-S02	0,021	S11-S04	0,037	S11-S05	0,030	S08-S02	0,151
S07-S01	0,022	S09-S05	0,041	S09-S03	0,033	S08-S11	0,151
S11-S02	0,026	S07-S04	0,045	S09-S02	0,037	S07-S01	0,167
S09-S03	0,030	S10-S03	0,050	S11-S03	0,068	S03-S01	0,192
S08-S04	0,050	S08-S02	0,056	S09-S04	0,074	S06-S03	0,240
S07-S03	0,052	S10-S06	0,090	S11-S02	0,074	S08-S05	0,240
S08-S05	0,056	S02-S04	0,090	S08-S06	0,082	S08-S09	0,240
S10-S04	0,056	S11-S05	0,098	S10-S01	0,082	S04-S02	0,258
S10-S05	0,061	S07-S05	0,106	S07-S01	0,094	S04-S11	0,258
S09-S02	0,061	S08-S11	0,128	S10-S05	0,128	S09-S01	0,338
S11-S04	0,068	S09-S03	0,128	S08-S10	0,139	S05-S01	0,338
S11-S05	0,074	S10-S01	0,139	S11-S04	0,139	S10-S11	0,361
S06-S01	0,074	S01-S04	0,151	S06-S01	0,139	S10-S02	0,361
S07-S02	0,094	S09-S06	0,207	S07-S05	0,139	S06-S10	0,384
S09-S04	0,139	S02-S05	0,207	S06-S05	0,207	S04-S05	0,384
S09-S05	0,151	S08-S07	0,220	S09-S06	0,223	S04-S09	0,384
S06-S03	0,164	S10-S02	0,223	S08-S07	0,228	S06-S07	0,403
S07-S04	0,186	S06-S04	0,223	S10-S03	0,240	S08-S03	0,408
S07-S05	0,199	S07-S03	0,251	S07-S03	0,243	S07-S02	0,448
S08-S06	0,207	S08-S09	0,258	S10-S02	0,258	S07-S11	0,448
S10-S06	0,223	S11-S03	0,258	S07-S02	0,259	S02-S01	0,486
S11-S06	0,258	S09-S01	0,296	S09-S10	0,338	S11-S01	0,486
S05-S01	0,258	S01-S05	0,317	S11-S06	0,361	S06-S04	0,514
S06-S02	0,277	S03-S04	0,338	S06-S03	0,361	S10-S05	0,514
S04-S01	0,277	S07-S06	0,360	S04-S01	0,361	S10-S09	0,514
S09-S06	0,433	S11-S06	0,384	S06-S02	0,384	S03-S11	0,542
S05-S03	0,459	S10-S11	0,408	S07-S04	0,392	S03-S02	0,542
S07-S06	0,484	S09-S02	0,433	S08-S11	0,408	S08-S07	0,599
S06-S04	0,486	S06-S05	0,433	S10-S04	0,408	S07-S05	0,599
S04-S03	0,486	S02-S03	0,459	S09-S07	0,460	S07-S09	0,599
S02-S01	0,486	S07-S01	0,471	S04-S05	0,486	S08-S10	0,602
S06-S05	0,514	S08-S10	0,486	S11-S10	0,514	S04-S03	0,602
S08-S09	0,632	S11-S01	0,514	S02-S01	0,542	S06-S08	0,728
S10-S09	0,663	S10-S07	0,546	S06-S04	0,572	S03-S09	0,728
S05-S02	0,663	S03-S05	0,602	S03-S01	0,572	S03-S05	0,728
S08-S07	0,669	S07-S02	0,627	S08-S09	0,602	S08-S04	0,761
S04-S02	0,695	S02-S06	0,632	S11-S07	0,640	S10-S03	0,761
S03-S01	0,695	S01-S03	0,632	S02-S05	0,695	S05-S11	0,794
S10-S07	0,697	S10-S09	0,663	S07-S06	0,726	S05-S02	0,794
S11-S09	0,728	S05-S04	0,663	S04-S03	0,728	S09-S02	0,794
S11-S07	0,755	S09-S11	0,695	S03-S05	0,728	S09-S11	0,794
S02-S03	0,761	S11-S02	0,695	S04-S02	0,761	S04-S07	0,800
S08-S11	0,896	S02-S01	0,794	S09-S11	0,761	S04-S10	0,828
S10-S11	0,931	S06-S03	0,794	S10-S06	0,794	S07-S03	0,830
S08-S10	0,965	S01-S06	0,828	S05-S01	0,828	S10-S07	0,953
S05-S04	0,965	S09-S07	0,830	S07-S10	0,907	S05-S09	1,000
S07-S09	1,000	S07-S11	0,892	S02-S03	0,965	S02-S11	1,000

DkEIL1 Comp.	Sig.	*DkEIL3* Comp.	Sig.	*DkPSY* Comp.	Sig.	*DkXTH9* Comp.	Sig.	*DkETR2* Comp.	Sig.
S08-S05	0,002	S09-S05	0,005	S02-S11	0,006	S07-S05	0,013	S08-S01	0,002
S10-S05	0,030	S08-S05	0,010	S04-S11	0,009	S02-S05	0,017	S06-S01	0,003
S08-S06	0,033	S10-S05	0,024	S03-S11	0,010	S07-S11	0,020	S09-S01	0,006
S09-S05	0,056	S09-S04	0,030	S02-S09	0,021	S02-S11	0,026	S08-S03	0,013
S08-S01	0,074	S08-S04	0,056	S01-S11	0,021	S07-S09	0,043	S08-S04	0,019
S08-S04	0,074	S09-S02	0,082	S06-S11	0,024	S02-S09	0,061	S08-S02	0,021
S08-S02	0,074	S09-S03	0,090	S04-S09	0,030	S07-S04	0,094	S06-S03	0,021
S08-S11	0,107	S09-S06	0,090	S03-S09	0,033	S07-S10	0,102	S06-S04	0,030
S08-S03	0,117	S09-S01	0,098	S05-S11	0,037	S01-S05	0,107	S06-S02	0,033
S03-S05	0,117	S11-S05	0,098	S02-S10	0,056	S07-S08	0,129	S08-S10	0,037
S11-S05	0,128	S10-S04	0,107	S01-S09	0,061	S02-S04	0,139	S09-S03	0,037
S07-S05	0,134	S08-S02	0,139	S06-S09	0,068	S03-S05	0,139	S09-S04	0,050
S02-S05	0,177	S08-S03	0,151	S02-S08	0,074	S02-S10	0,151	S09-S02	0,056
S04-S05	0,177	S08-S06	0,151	S04-S10	0,074	S01-S11	0,151	S06-S10	0,056
S01-S05	0,177	S07-S05	0,155	S03-S10	0,082	S07-S06	0,186	S08-S05	0,068
S08-S07	0,192	S08-S01	0,164	S04-S08	0,098	S02-S08	0,192	S11-S01	0,082
S08-S09	0,223	S09-S11	0,240	S05-S09	0,098	S03-S11	0,192	S07-S01	0,083
S10-S06	0,240	S10-S02	0,240	S02-S07	0,102	S06-S05	0,192	S09-S10	0,090
S06-S05	0,317	S01-S05	0,240	S03-S08	0,107	S07-S03	0,243	S06-S05	0,098
S08-S10	0,338	S10-S03	0,258	S04-S07	0,129	S06-S11	0,258	S09-S05	0,151
S09-S06	0,361	S10-S06	0,258	S01-S10	0,139	S02-S06	0,277	S08-S11	0,164
S10-S04	0,408	S03-S05	0,258	S03-S07	0,139	S01-S09	0,277	S05-S01	0,192
S10-S02	0,408	S06-S05	0,258	S06-S10	0,151	S08-S05	0,277	S06-S11	0,223
S10-S01	0,408	S09-S07	0,267	S01-S08	0,177	S07-S01	0,293	S07-S03	0,251
S10-S11	0,514	S10-S01	0,277	S06-S08	0,192	S03-S09	0,338	S11-S03	0,277
S10-S03	0,542	S02-S05	0,277	S05-S10	0,207	S10-S05	0,338	S08-S07	0,284
S07-S06	0,546	S11-S04	0,317	S01-S07	0,213	S02-S03	0,361	S10-S01	0,296
S09-S02	0,572	S08-S11	0,361	S06-S07	0,228	S08-S11	0,361	S07-S04	0,302
S09-S01	0,572	S08-S07	0,381	S05-S08	0,258	S04-S05	0,361	S09-S11	0,317
S09-S04	0,572	S07-S04	0,403	S05-S07	0,293	S02-S01	0,433	S07-S02	0,321
S03-S06	0,572	S04-S05	0,514	S08-S11	0,338	S06-S09	0,433	S11-S04	0,338
S11-S06	0,602	S10-S11	0,542	S10-S11	0,408	S10-S11	0,433	S06-S07	0,360
S10-S07	0,654	S10-S07	0,546	S07-S11	0,414	S04-S11	0,459	S11-S02	0,361
S09-S11	0,695	S09-S10	0,572	S02-S05	0,514	S01-S04	0,486	S02-S01	0,408
S09-S03	0,728	S11-S02	0,572	S04-S05	0,602	S01-S10	0,514	S07-S10	0,425
S04-S06	0,728	S11-S03	0,602	S08-S09	0,602	S03-S04	0,572	S04-S01	0,433
S02-S06	0,728	S11-S06	0,602	S02-S06	0,632	S08-S09	0,572	S09-S07	0,471
S01-S06	0,728	S01-S04	0,602	S03-S05	0,632	S01-S08	0,602	S11-S10	0,486
S07-S01	0,770	S11-S01	0,632	S02-S01	0,663	S03-S10	0,602	S05-S03	0,514
S07-S04	0,770	S06-S04	0,632	S09-S11	0,663	S09-S05	0,602	S03-S01	0,514
S07-S02	0,770	S03-S04	0,632	S07-S09	0,669	S10-S09	0,663	S07-S05	0,572
S10-S09	0,794	S07-S02	0,654	S10-S09	0,695	S03-S08	0,695	S05-S04	0,602
S03-S02	0,828	S02-S04	0,663	S04-S06	0,728	S06-S04	0,695	S05-S02	0,632
S03-S01	0,828	S07-S03	0,683	S04-S01	0,761	S04-S09	0,695	S11-S05	0,663
S03-S04	0,828	S07-S06	0,683	S03-S06	0,761	S07-S02	0,726	S08-S09	0,695
S09-S07	0,830	S07-S01	0,712	S03-S01	0,794	S06-S10	0,728	S10-S03	0,695
S11-S04	0,862	S08-S10	0,761	S01-S05	0,828	S09-S11	0,728	S05-S10	0,794
S11-S01	0,862	S09-S08	0,794	S02-S03	0,862	S01-S06	0,761	S10-S04	0,794
S11-S02	0,862	S01-S02	0,931	S06-S05	0,862	S06-S08	0,828	S10-S02	0,828
S07-S11	0,892	S11-S07	0,953	S02-S04	0,896	S03-S06	0,862	S06-S09	0,828
S07-S03	0,922	S01-S06	0,965	S08-S10	0,896	S08-S04	0,862	S07-S11	0,861
S03-S11	0,965	S01-S03	0,965	S07-S10	0,938	S11-S05	0,862	S08-S06	0,862
S01-S04	1,000	S03-S02	0,965	S04-S03	0,965	S01-S03	0,896	S02-S03	0,862
S01-S02	1,000	S06-S02	0,965	S01-S06	0,965	S08-S10	0,896	S04-S03	0,896
S02-S04	1,000	S03-S06	1,000	S08-S07	0,969	S10-S04	0,965	S02-S04	0,965

ROJO BRILLANTE

Test: Kruskal-Wallis (p<0.05)

TRAIT / GENE	Sig.
WEIGHT	0,000
CI	0,000
FIRNESS	0,000
DkERF16	0,011
DkERF8	0,001
DkERF18	0,001
DkACO1	0,043
DkACO2	0,003
DkACS2	0,045
DkERS1	0,005
DkETR3	0,010
DkEIL1	0,151
DkEIL3	0,076
DkPSY	0,009
DkXTH9	0,043
DkETR2	0,004

Pairwise Comparisons (p<0.05)

WEIGHT		CI		FIRMNESS	
Comp.	Sig.	Comp.	Sig.	Comp.	Sig.
S1-S10	0,000	S2-S12	0,000	S13-S1	0,000
S1-S8	0,000	S1-S12	0,000	S13-S2	0,000
S1-S7	0,000	S2-S11	0,000	S13-S3	0,000
S2-S10	0,000	S1-S11	0,000	S12-S1	0,000
S1-S9	0,000	S1-S13	0,000	S12-S2	0,000
S3-S10	0,000	S2-S13	0,000	S11-S1	0,000
S2-S8	0,000	S3-S12	0,000	S12-S3	0,000
S1-S11	0,000	S1-S10	0,000	S11-S2	0,000
S2-S7	0,000	S3-S11	0,000	S11-S3	0,000
S3-S8	0,000	S2-S10	0,000	S10-S1	0,000
S3-S7	0,000	S4-S12	0,000	S13-S4	0,000
S2-S9	0,000	S4-S11	0,000	S10-S2	0,000
S1-S6	0,000	S1-S9	0,000	S10-S3	0,000
S3-S9	0,001	S2-S9	0,000	S12-S4	0,000
S2-S11	0,001	S3-S13	0,001	S13-S5	0,001
S3-S11	0,001	S5-S12	0,001	S11-S4	0,001
S4-S10	0,002	S5-S11	0,001	S9-S1	0,001
S2-S6	0,002	S3-S10	0,001	S9-S2	0,001
S1-S12	0,003	S4-S13	0,002	S9-S3	0,001
S3-S6	0,003	S1-S8	0,003	S10-S4	0,001
S4-S8	0,005	S4-S10	0,003	S12-S5	0,003
S13-S10	0,005	S2-S8	0,003	S11-S5	0,004
S4-S7	0,006	S6-S12	0,004	S8-S1	0,004
S1-S5	0,009	S3-S9	0,004	S13-S6	0,004
S13-S8	0,010	S6-S11	0,005	S8-S2	0,006
S4-S9	0,010	S1-S7	0,007	S8-S3	0,006
S13-S7	0,012	S2-S7	0,009	S10-S5	0,007
S2-S12	0,015	S4-S9	0,011	S9-S4	0,010
S3-S12	0,019	S5-S13	0,011	S13-S7	0,012
S13-S9	0,020	S5-S10	0,016	S12-S6	0,018
S4-S11	0,022	S7-S12	0,018	S7-S1	0,023
S2-S5	0,038	S7-S11	0,021	S11-S6	0,025
S13-S11	0,040	S1-S6	0,030	S7-S3	0,032
S4-S6	0,042	S3-S8	0,035	S7-S2	0,033
S3-S5	0,044	S2-S6	0,038	S10-S6	0,038
S5-S10	0,054	S6-S13	0,038	S8-S4	0,040
S13-S6	0,072	S8-S12	0,039	S9-S5	0,042
S1-S13	0,083	S5-S9	0,044	S12-S7	0,042
S5-S8	0,087	S8-S11	0,045	S6-S1	0,051
S5-S7	0,105	S6-S10	0,050	S13-S8	0,053
S12-S10	0,113	S3-S7	0,070	S11-S7	0,055
S1-S4	0,134	S4-S8	0,070	S6-S3	0,069
S4-S12	0,145	S1-S5	0,087	S6-S2	0,074
S5-S9	0,148	S2-S5	0,108	S10-S7	0,080
S12-S8	0,170	S6-S9	0,119	S8-S5	0,128
S12-S7	0,199	S7-S13	0,122	S7-S4	0,141
S13-S12	0,222	S4-S7	0,131	S12-S8	0,146
S5-S11	0,242	S7-S10	0,152	S13-S9	0,155
S2-S13	0,244	S9-S12	0,182	S9-S6	0,155
S3-S13	0,258	S3-S6	0,199	S11-S8	0,180
S4-S5	0,263	S5-S8	0,199	S5-S1	0,182
S12-S9	0,268	S9-S11	0,204	S5-S3	0,230
S6-S10	0,314	S8-S13	0,213	S10-S8	0,242
S5-S6	0,358	S1-S4	0,237	S5-S2	0,251
S2-S4	0,359	S8-S10	0,258	S6-S4	0,252
S3-S4	0,372	S2-S4	0,290	S9-S7	0,274
S13-S5	0,379	S7-S9	0,302	S7-S5	0,345
S12-S11	0,406	S4-S6	0,327	S12-S9	0,349
S6-S8	0,428	S5-S7	0,327	S8-S6	0,365
S11-S10	0,450	S10-S12	0,352	S11-S9	0,410
S6-S7	0,481	S1-S3	0,379	S4-S1	0,421
S1-S2	0,525	S10-S11	0,385	S13-S10	0,443
S1-S3	0,546	S3-S5	0,406	S4-S3	0,501
S12-S6	0,563	S6-S8	0,406	S10-S9	0,513
S11-S8	0,589	S13-S12	0,414	S6-S5	0,538
S6-S9	0,597	S13-S11	0,450	S4-S2	0,548
S9-S10	0,633	S2-S3	0,457	S13-S11	0,550
S11-S7	0,651	S8-S9	0,466	S8-S7	0,563
S5-S12	0,734	S4-S5	0,597	S5-S4	0,597
S7-S10	0,763	S6-S7	0,597	S9-S8	0,606
S11-S9	0,782	S9-S13	0,606	S13-S12	0,628
S9-S8	0,792	S5-S6	0,651	S7-S6	0,744
S6-S11	0,801	S9-S10	0,687	S12-S10	0,777
S4-S13	0,811	S3-S4	0,763	S2-S1	0,814
S8-S10	0,831	S7-S8	0,763	S11-S10	0,865
S9-S7	0,860	S1-S2	0,865	S3-S1	0,895
S7-S8	0,930	S10-S13	0,910	S12-S11	0,910
S3-S2	0,993	S11-S12	0,950	S2-S3	0,922

DkERF16		*DkERF8*		*DkERF18*	
Comp.	Sig.	Comp.	Sig.	Comp.	Sig.
S08-S13	0,000	S02-S13	0,000	S09-S02	0,000
S08-S12	0,002	S02-S11	0,001	S07-S02	0,000
S03-S13	0,004	S03-S13	0,001	S08-S02	0,001
S05-S13	0,008	S01-S13	0,002	S09-S03	0,001
S08-S11	0,008	S02-S12	0,004	S09-S01	0,002
S09-S13	0,012	S04-S13	0,005	S07-S03	0,003
S03-S12	0,013	S03-S11	0,005	S07-S01	0,004
S08-S04	0,016	S01-S11	0,008	S08-S03	0,005
S08-S10	0,020	S02-S10	0,008	S08-S01	0,006
S05-S12	0,022	S05-S13	0,008	S05-S02	0,009
S07-S13	0,025	S03-S12	0,013	S06-S02	0,009
S08-S06	0,030	S02-S09	0,016	S09-S04	0,010
S09-S12	0,033	S04-S11	0,017	S09-S13	0,015
S03-S11	0,036	S01-S12	0,019	S07-S04	0,021
S05-S11	0,054	S03-S10	0,024	S09-S11	0,028
S08-S02	0,058	S05-S11	0,029	S07-S13	0,030
S07-S12	0,059	S01-S10	0,035	S08-S04	0,030
S09-S11	0,074	S03-S09	0,040	S05-S01	0,037
S03-S04	0,075	S04-S12	0,042	S06-S01	0,037
[illegible]	[illegible]	[illegible]	[illegible]	[illegible]	[illegible]

DkACO1		*DkACO2*		*DkACS2*		*DkERS1*		*DkETR3*		*DkEIL1*		*DkEIL3*		*DkPSY*		*DkXTH9*		*DkETR2*	
Comp.	Sig.	Comp.	Sig.	Comp.	Sig.	Comp.	Sig.	Comp.	Sig.	Comp.	Sig.	Comp.	Sig.	Comp.	Sig.	Comp.	Sig.	Comp.	Sig.
[illegible]	[illegible]	[illegible]	[illegible]	[illegible]	[illegible]	[illegible]	[illegible]	[illegible]	[illegible]	[illegible]	[illegible]	[illegible]	[illegible]	[illegible]	[illegible]	[illegible]	[illegible]	[illegible]	[illegible]

ISAHAYA

Test: Kruskal-Wallis (p<0.05)

TRAIT / GENE	Sig.
WEIGHT	0,000
CI	0,000
FIRNESS	0,000
DkERF16	0,056
DkERF8	0,003
DkERF18	0,003
DkACO1	0,009
DkACO2	0,003
DkACS2	0,001
DkERS1	0,006
DkETR3	0,012
DkEIL1	0,004
DkEIL3	0,021
DkPSY	0,001
DkXTH9	0,249
DkETR2	0,069

Pairwise Comparisons (p<0.05)

WEIGHT		CI		FIRMNESS	
Comp.	Sig.	Comp.	Sig.	Comp.	Sig.
S1-S10	0,000	S1-S13	0,000	S13-S1	0,000
S1-S13	0,000	S2-S13	0,000	S12-S1	0,000
S1-S7	0,000	S1-S12	0,000	S13-S2	0,000
S2-S10	0,000	S2-S12	0,000	S11-S1	0,000
S2-S13	0,000	S3-S13	0,000	S13-S3	0,000
S2-S7	0,000	S3-S12	0,000	S12-S2	0,000
S3-S10	0,000	S1-S11	0,000	S13-S4	0,000
S5-S10	0,000	S4-S13	0,000	S10-S1	0,000
S3-S13	0,000	S2-S11	0,000	S11-S2	0,000
S3-S7	0,000	S4-S12	0,000	S7-S1	0,000
S5-S13	0,000	S1-S10	0,000	S12-S3	0,000
S5-S7	0,000	S2-S10	0,000	S12-S4	0,001
S1-S12	0,001	S3-S11	0,000	S9-S1	0,001
S1-S11	0,001	S5-S13	0,001	S13-S5	0,001
S4-S10	0,001	S1-S9	0,001	S11-S3	0,002
S1-S6	0,001	S4-S11	0,001	S13-S8	0,002
S4-S13	0,001	S3-S10	0,001	S10-S2	0,002
S4-S7	0,001	S5-S12	0,001	S11-S4	0,003
S1-S9	0,001	S2-S9	0,001	S7-S2	0,003
S2-S12	0,004	S4-S10	0,002	S13-S6	0,005
S1-S8	0,004	S6-S13	0,003	S12-S5	0,007
S2-S11	0,005	S6-S12	0,004	S9-S2	0,010
S2-S6	0,007	S1-S8	0,004	S6-S1	0,010
S2-S9	0,009	S3-S9	0,005	S12-S8	0,011
S2-S8	0,023	S2-S8	0,007	S10-S3	0,012
S3-S12	0,029	S5-S11	0,008	S7-S3	0,015
S3-S11	0,030	S1-S7	0,010	S10-S4	0,019
S5-S12	0,031	S4-S9	0,011	S8-S1	0,023
S5-S11	0,032	S2-S7	0,014	S7-S4	0,024
S8-S10	0,038	S7-S13	0,015	S12-S6	0,025
S3-S6	0,039	S5-S10	0,022	S11-S5	0,026
S5-S6	0,042	S7-S12	0,022	S13-S9	0,034
S3-S9	0,050	S3-S8	0,023	S5-S1	0,034
S5-S9	0,053	S6-S11	0,026	S11-S8	0,037
S8-S13	0,059	S8-S13	0,029	S9-S3	0,038
S8-S7	0,061	S8-S12	0,042	S9-S4	0,057
S4-S12	0,064	S3-S7	0,044	S6-S2	0,067
S4-S11	0,066	S4-S8	0,047	S11-S6	0,074
S9-S10	0,080	S1-S6	0,047	S13-S7	0,076
S4-S6	0,084	S6-S10	0,061	S13-S10	0,092
S6-S10	0,098	S2-S6	0,065	S10-S5	0,112
S3-S8	0,101	S5-S9	0,072	S8-S2	0,126
S4-S9	0,104	S4-S7	0,082	S12-S9	0,127
S5-S8	0,106	S7-S11	0,101	S7-S5	0,134
S9-S13	0,118	S9-S13	0,101	S10-S8	0,148
S9-S7	0,120	S1-S5	0,122	S5-S2	0,167
S11-S10	0,123	S9-S12	0,136	S6-S3	0,174
S12-S10	0,127	S3-S6	0,151	S7-S8	0,176
S1-S4	0,137	S2-S5	0,159	S4-S1	0,183
S6-S13	0,143	S8-S11	0,164	S6-S4	0,233
S6-S7	0,146	S6-S9	0,168	S12-S7	0,238
S11-S13	0,176	S7-S10	0,196	S3-S1	0,247
S11-S7	0,180	S5-S8	0,210	S10-S6	0,248
S12-S13	0,180	S4-S6	0,248	S9-S5	0,251
S12-S7	0,184	S10-S13	0,253	S12-S10	0,277
S4-S8	0,192	S8-S10	0,297	S11-S9	0,283
S1-S5	0,244	S3-S5	0,311	S8-S3	0,285
S1-S3	0,254	S5-S7	0,314	S7-S6	0,288
S2-S4	0,355	S10-S12	0,320	S13-S11	0,294
S2-S5	0,547	S9-S11	0,398	S9-S8	0,314
S1-S2	0,558	S6-S8	0,405	S5-S3	0,355
S2-S3	0,564	S7-S9	0,427	S8-S4	0,368
S8-S12	0,585	S11-S13	0,427	S2-S1	0,444
S8-S11	0,593	S1-S4	0,433	S5-S4	0,449
S8-S6	0,673	S4-S5	0,464	S11-S7	0,467
S3-S4	0,737	S2-S4	0,518	S9-S6	0,475
S8-S9	0,747	S11-S12	0,518	S11-S10	0,526
S5-S4	0,756	S6-S7	0,559	S13-S12	0,551
S9-S12	0,823	S8-S9	0,585	S4-S2	0,551
S9-S11	0,833	S9-S10	0,619	S12-S11	0,650
S7-S10	0,842	S1-S3	0,621	S10-S9	0,659
S13-S10	0,852	S5-S6	0,673	S6-S5	0,664
S6-S12	0,901	S2-S3	0,722	S3-S2	0,673
S6-S11	0,911	S10-S11	0,728	S7-S9	0,728
S9-S6	0,921	S3-S4	0,780	S6-S8	0,770
S3-S5	0,980	S7-S8	0,804	S4-S3	0,867
S11-S12	0,990	S12-S13	0,881	S8-S5	0,886
S7-S13	0,990	S1-S2	0,886	S10-S7	0,926

DkERF16		*DkERF8*		*DkERF18*	
Comp.	Sig.	Comp.	Sig.	Comp.	Sig.
S09-S01	0,002	S05-S13	0,001	S09-S03	0,001
S06-S01	0,005	S05-S11	0,002	S09-S02	0,001
S09-S11	0,007	S02-S13	0,002	S08-S03	0,001
S04-S01	0,008	S02-S11	0,003	S08-S02	0,002
S09-S07	0,018	S06-S13	0,007	S09-S01	0,003
S06-S11	0,018	S01-S13	0,007	S08-S01	0,005
S04-S11	0,026	S05-S12	0,008	S06-S03	0,009
S05-S01	0,035	S05-S10	0,008	S09-S04	0,011
S10-S01	0,038	S04-S13	0,009	S06-S02	0,011
S06-S07	0,041	S06-S11	0,012	S09-S11	0,013
S13-S01	0,041	S01-S11	0,013	S08-S04	0,020
S03-S01	0,045	S02-S12	0,015	S08-S11	0,024
S09-S02	0,053	S02-S10	0,015	S13-S03	0,024
S04-S07	0,058	S04-S11	0,016	S06-S01	0,029
S05-S11	0,092	S08-S13	0,022	S13-S02	0,029
S10-S11	0,100	S08-S11	0,038	S09-S07	0,032
S12-S01	0,100	S03-S13	0,041	S12-S03	0,032
S06-S02	0,107	S06-S10	0,045	S12-S02	0,038
S13-S11	0,107	S06-S12	0,045	S09-S10	0,049
S09-S08	0,115	S01-S12	0,049	S08-S07	0,053
S03-S11	0,115	S01-S10	0,049	S13-S01	0,068
S08-S01	0,124	S07-S13	0,049	S09-S05	0,079
S09-S12	0,142	S04-S10	0,058	S08-S10	0,079
S04-S02	0,142	S04-S12	0,058	S06-S04	0,086
[illegible]	[illegible]	[illegible]	[illegible]	[illegible]	[illegible]

DkACO1		*DkACO2*		*DkACS2*		*DkERS1*		*DkETR3*		*DkEIL1*		*DkEIL3*		*DkPSY*		*DkXTH9*		*DkETR2*	
Comp.	Sig.	Comp.	Sig.	Comp.	Sig.	Comp.	Sig.	Comp.	Sig.	Comp.	Sig.	Comp.	Sig.	Comp.	Sig.	Comp.	Sig.	Comp.	Sig.
[illegible]	[illegible]	[illegible]	[illegible]	[illegible]	[illegible]	[illegible]	[illegible]	[illegible]	[illegible]	[illegible]	[illegible]	[illegible]	[illegible]	[illegible]	[illegible]	[illegible]	[illegible]	[illegible]	[illegible]

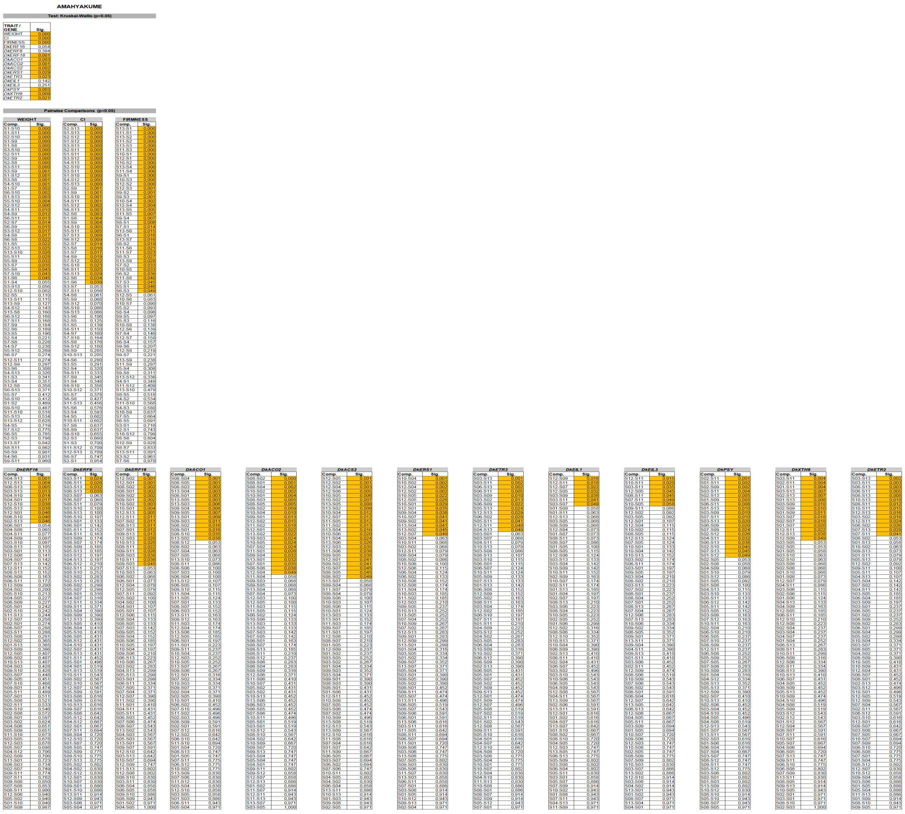

AMAHYAKUME

Test: Kruskal-Wallis (p<0.05)

TRAIT / GENE	Sig.
WEIGHT	0,000
CI	0,000
FIRNESS	0,000
DkERF16	0,054
DkERF8	0,394
DkERF18	0,001
DkACO1	0,003
DkACO2	0,001
DkACS2	0,002
DkERS1	0,029
DkETR3	0,023
DkEIL1	0,142
DkEIL3	0,251
DkPSY	0,003
DkXTH9	0,009
DkETR2	0,021

Pairwise Comparisons (p<0.05)

[illegible]

Name	Forward primer (5' to 3')	Reverse primer (5' to 3')	Source
DkACO1	TGGCAATGATGCTGTTATCTATC	CGAACTATTACAAATAACATGTGTC	Ortiz et al., 2006. doi: https://doi.org/10.2503/jjshs.75.178
DkACO2	CAGCGACGCAGTGATTTATCCAG	CAGAGGGCTTGGCTTAGACTGTGGC	Ortiz et al., 2006. doi: https://doi.org/10.2503/jjshs.75.178
DkACS2	TCCAACATCAGAGTAAAGGGCT	GTCTAGGTTGCAGTCCATTTCC	Ortiz et al., 2006. doi: https://doi.org/10.2503/jjshs.75.178
DkEIL1	GCATGGAGAGGGTTAAAGATAGG	GACACTGCAGGAACTCACATGTAT	This study
DkEIL3	TCAGAGGAAGTACCCCTTAGAGAA	TATAAGGAGGACTCTGACCTTTGG	This study
DkERF8	ATCTGGAAGGGGGACAATTC	AGAGTAGCGCGGCAAAATTA	Park et al., 2017. J Am Pom Soc. (APS) 71:103-111
DkERF16	TCAGGCTATGTGATCTACCTCTC	CACTACTCACAGAGGGGTAAAAAT	Min et al., 2014. doi:https://doi.org/10.1371/journal.pone.0097043
DkERF18	GACGTCGTCTTCCTCGTCTTA	GCCTATTTTAACACTCCACACTG	Min et al., 2014. doi:https://doi.org/10.1371/journal.pone.0097043
DkERS1	CAGAACAGAGGGTCATGATAGAGA	CTTCTCATCACCAACAGCATAGAC	This study
DkETR2	GCTCCTTTACTTCGTTAGTTGCTC	CCTTTCATCTAATGCTAGCCCTC	This study
DkETR3	ATCGAGCTTCTCTACTTCGTTAGC	GTTTCAGTTAATGGTAGCCCTCAC	This study
DkPSY	AACTATGGCTCTTTCTATGC	AACTTTCTGCTCCGATGA	Zhao et al., 2011. doi:https://doi.org/10.1007/s11105-010-0272-3
DkTUA	AGCCCTCAAGTATGATGG	GCCTACAGCAGCATTAAC	Wang et al., 2016. doi:https://doi.org/10.1371/journal.pone.0160885
DkXTH9	AGGCGATGAGGGTATATGCAAG	AAATCCGACTCTTTTGCAAGCC	This study

Table S2. List of gene-specific primers used for real-time quantitative PCR expression analysis.
